# Phage Targeting Neonatal Meningitis *E. coli* K1 In Vitro in the Intestinal Microbiota of Pregnant Donors and Impact on Bacterial Populations

**DOI:** 10.3390/ijms241310580

**Published:** 2023-06-24

**Authors:** Céline Antoine, Fanny Laforêt, Elizabeth Goya-Jorge, Irma Gonza, Sarah Lebrun, Caroline Douny, Jean-Noël Duprez, Abdoulaye Fall, Bernard Taminiau, Marie-Louise Scippo, Georges Daube, Damien Thiry, Véronique Delcenserie

**Affiliations:** 1 Laboratory of Veterinary Bacteriology, Department of Infectious and Parasitic Diseases, FARAH and Faculty of Veterinary Medicine, University of Liège, 4000 Liège, Belgium; celine.antoine@uliege.be (C.A.); fanny.laforet@uliege.be (F.L.); jean-noel.duprez@uliege.be (J.-N.D.); damien.thiry@uliege.be (D.T.); 2 Laboratory of Food Quality Management, Food Science Department, FARAH and Faculty of Veterinary Medicine, University of Liège, 4000 Liège, Belgium; egoya@uliege.be (E.G.-J.); iegonza@uliege.be (I.G.); sarah.lebrun@uliege.be (S.L.); 3 Laboratory of Food Analysis, Department of Food Sciences, FARAH and Faculty of Veterinary Medicine, University of Liège, 4000 Liège, Belgium; cdouny@uliege.be (C.D.); mlscippo@uliege.be (M.-L.S.); 4 FoodChain ID Genomics, En Hayeneux 62, 4040 Herstal, Belgium; abdoulaye.fall@foodchainid.com; 5 Laboratory of Microbiology, Department of Food Sciences, FARAH and Faculty of Veterinary Medicine, University of Liège, 4000 Liège, Belgium; bernard.taminiau@uliege.be (B.T.); georges.daube@uliege.be (G.D.)

**Keywords:** phage therapy, bacteriophage, *Escherichia coli* K1, SHIME^®^, intestinal microbiota, *Galleria mellonella*

## Abstract

*Escherichia coli* K1 is a leading cause of neonatal meningitis. The asymptomatic carriage of these strains in the maternal intestinal microbiota constitutes a risk of vertical transmission to the infant at birth. The aim of this work was to evaluate the efficacy of phage therapy against *E. coli* K1 in an intestinal environment and its impact on the intestinal microbiota. For this purpose, three independent experiments were conducted on the SHIME® system, the first one with only the phage vB_EcoP_K1_ULINTec4, the second experiment with only *E. coli* K1 and the last experiment with both *E. coli K1* and the phage. Microbiota monitoring was performed using metagenetics, qPCR, SCFA analysis and the induction of AhR. The results showed that phage vB_EcoP_K1_ULINTec4, inoculated alone, was progressively cleared by the system and replicates in the presence of its host. *E. coli* K1 persisted in the microbiota but decreased in the presence of the phage. The impact on the microbiota was revealed to be donor dependent, and the bacterial populations were not dramatically affected by vB_K1_ULINTec4, either alone or with its host. In conclusion, these experiments showed that the phage was able to infect the *E. coli* K1 in the system but did not completely eliminate the bacterial load.

## 1. Introduction

*Escherichia coli* is, with Group B streptococci (GBS), a leading cause of neonatal sepsis and meningitis worldwide. These infections can occur before or after 72 h of life, which differentiates between early-onset and late-onset. In pre-term newborns, *E. coli* is the most commonly isolated bacteria in neonatal meningitis cases [1,2,3]. The associated risks factors include low or very low birth weight, degree of prematurity, ruptured membranes and maternal peripartum infection [3,4,5]. Despite the use of effective antibiotics, neonatal meningitis has a mortality of 10% in developed countries and 40–50% in developing countries. Among the survivors, 50% develop devastating neurological sequelae and permanent disabilities [5,6,7]. The K1 capsular type is predominant in *E. coli* strains causing meningitis. This important virulence factor allows the bacteria to survive and multiply in and outside the gastrointestinal tract. In addition to being a major cause of meningitis and sepsis in newborns, *E. coli* K1 can cause urinary tract infections in humans and is also involved in several animal diseases, including avian colibacillosis [8,9]. Beta-lactam antibiotics are usually used in the empirical treatment of *E. coli* neonatal meningitis (ECNM), in combination with gentamicin [10]. However, antibiotic resistance due to the emergence of strains producing extended-spectrum beta-lactamase is becoming a concern, indicating the need to develop alternative treatments [10,11,12]. The pathogenesis of *E. coli* K1 associated meningitis is not fully understood. The early steps can include a gastro-intestinal colonization, followed by a bacterial translocation from the small intestine and the colon into the systemic circulation. The bacteriemia can lead to sepsis and/or meningitis by crossing the blood–brain barrier (BBB) [13,14]. Various factors are involved in the pathogenesis. The expression of a K1 capsule allows the passage and the intracellular survival of *E. coli* in human brain microvascular endothelial cells’ (HBMEC) vacuoles by modulating their maturation and by preventing fusion with lyzosomes [15,16]. In neonatal meningitis, this capsular type was linked to several specific serotypes: O18:K1:H7, O1:K1, O7:K1, O16:K1, O83:K1 and O45:K1:H7 [7,17]. Virulence factors *OmpA* and type 1 fimbriae have been associated with the attachment to the BBB, and *ibeA* and CNF1 with the bacterial invasion [15]. Several studies have focused on vaccinal prevention of ECNM [18,19]. It might be tempting to develop a vaccine targeting the K1 capsule but its structure, composed of α2,8-linked polysialic acid, is very similar to that of the human neural cell adhesion molecule, making its application problematic [20].

A perinatal vertical transmission from mother to infant is highly suspected to be an important first step in the onset of ECNM. Indeed, the presence of this bacteria in the neonate cerebrospinal fluid and the feces of both mother and infant is an indication that the maternal microbiota is a reservoir of ECNM strains. Extraintestinal *E. coli*, including *E. coli* K1, are frequently found as commensal bacteria in the gut microbiota of pregnant women and other healthy individuals. However, their ability to colonize other environments, like the vagina of pregnant women, and subsequently the gastrointestinal tract of newborns, are early steps for the development of ECNM. A rat model was able to reproduce some of the key features related to pathogenesis [13,14,21,22,23,24,25]. After the colonization of the gastrointestinal tract of newborn pups with *E. coli* K1, the colonizing bacteria could translocate from the lumen of the gastrointestinal tract to the bloodstream and reach the central nervous system [26]. This model has also been used to assess potential treatments and prevention solutions [27,28]. The SHIME^®^ (Simulator of Human Intestinal Microbial Ecosystem) (Prodigest, Ghent, Belgium) is a complex and dynamic in vitro gastro-intestinal model useful for long-term experiments. It allows the study of the behavior and interactions of bacteria, phages and gut microbiota within the digestive system. The SHIME^®^ model has shown promising results in several studies, highlighting its potential as a valuable tool in phage research [29,30,31,32]. 

Phage therapy is a promising alternative to antibiotics and has already been evaluated to treat ECNM through systemic delivery [33,34,35]. The efficacy of vB_EcoP_K1_ULINTec4 (K1_ULINTec4), a K1-dependent phage targeting *E. coli* K1, showed an improvement in its survival on *E. coli*-infected *Galleria mellonella* larvae [33]. In this study, we investigated the potential of phage therapy and the impact of this treatment on bacterial populations and productions in the prevention of neonatal meningitis by specifically targeting *E. coli* K1 in the gut microbiota of pregnant women, using the SHIME^®^ model. Three different experimental conditions were designed for assessment: the persistence and the effects of the phage alone, the effect of *E. coli* K1 alone and the dynamics and effects of the phage treatment in the presence of *E. coli* K1 on the intestinal microbiota. 

## 2. Results

### 2.1. Determination of the Optimal Phage Concentration in the Galleria mellonella Model

The preliminary experiment allowed the determination of the optimal dose of the inoculation of *E. coli* K1 (C5) in the model, enabling a lethality between 90 and 100%. This concentration, 10^6^ CFU/10 µL, was used for the main experiment.

**Figure 1:**
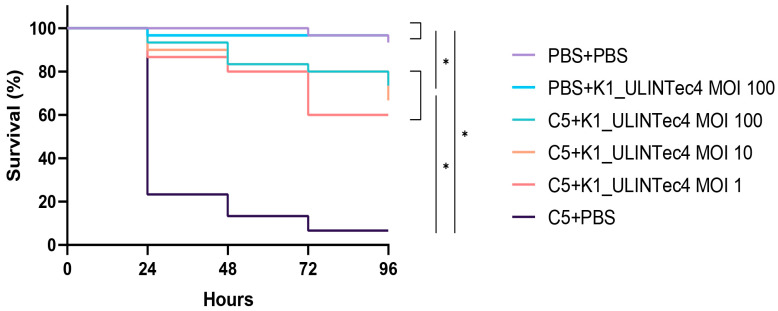
Survival curves of the main experiment assessing different MOIs of the phage K1_ULINTec4 in the *G. mellonella* model. C5: *E. coli* K1 strain C5, PBS: phosphate-buffered saline. Statistical significance is indicated as *p* < 0.05 (*).

The survival curves of the main experiment showed that infected phage-treated larvae had significantly higher survival rates than infected, untreated larvae. No significant differences were identified between the different treatment multiplicities of infection (MOIs). Treated larvae had significantly lower survival rates than uninfected controls (PBS + PBS and PBS + K1_ULINTec4 MOI 100). The survival curves are presented in Figure 1. Based on these results, an MOI of 1 was chosen for the experiments conducted in the SHIME model.

### 2.2. Triple-SHIME Experiments

The results related to the concentration of K1_ULINTec4 and *E. coli* K1 that were added to the SHIME systems of the three different donors were analyzed together. However, due to the large inter-individual variability between the microbiota of the donors, results related to intestinal microbiota (metagenetics, qPCR, SCFA and AhR) were analyzed separately, except for alpha diversity results.

#### 2.2.1. Persistence and Evolution of the Concentration of K1_ULINTec4 

**Figure 2:**
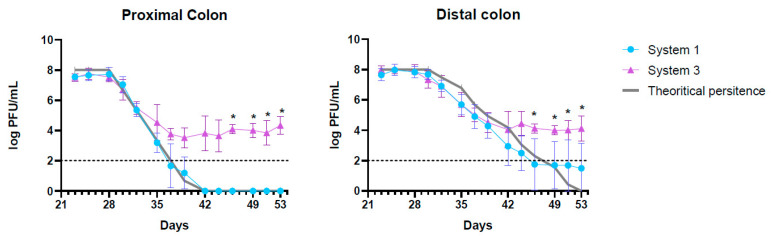
Graphical representation of the theoretical K1_ULINTec4 concentrations and those measured in systems 1 (phage alone) and 3 (bacteria + phage) for the proximal and distal colons. The replicates represent the mean results of the three separate donors with SD. The dotted line represents the limit of quantification. Statistical significance is indicated as *p* < 0.05 (*).

The elimination of active K1_ULINTec4 from system 1 (inoculated with phage alone) was close to the theoretical calculated elimination of inert molecules. This reflects a good stability of the phage in the system, for several days at 37 °C and despite pH variations between the proximal and distal colons. However, prolonged phage persistence above the limit of quantification was detected for donor 2 at the end of the run (Figure S1). In system 3 (inoculated with both bacteria and phage), the phage concentration followed the same trend at the beginning of the run, and then reached a stabilization at around 10^4^ PFU/mL. At day 46, this difference in phage persistence between systems 1 and 3 was significant in both proximal and distal colons (*p* = 0.02). The curves representing the evolution of the theoretical phage concentrations and those measured in systems 1 and 3 are presented in Figure 2.

#### 2.2.2. Evolution of *E. coli* K1 Concentrations 

**Figure 3:**
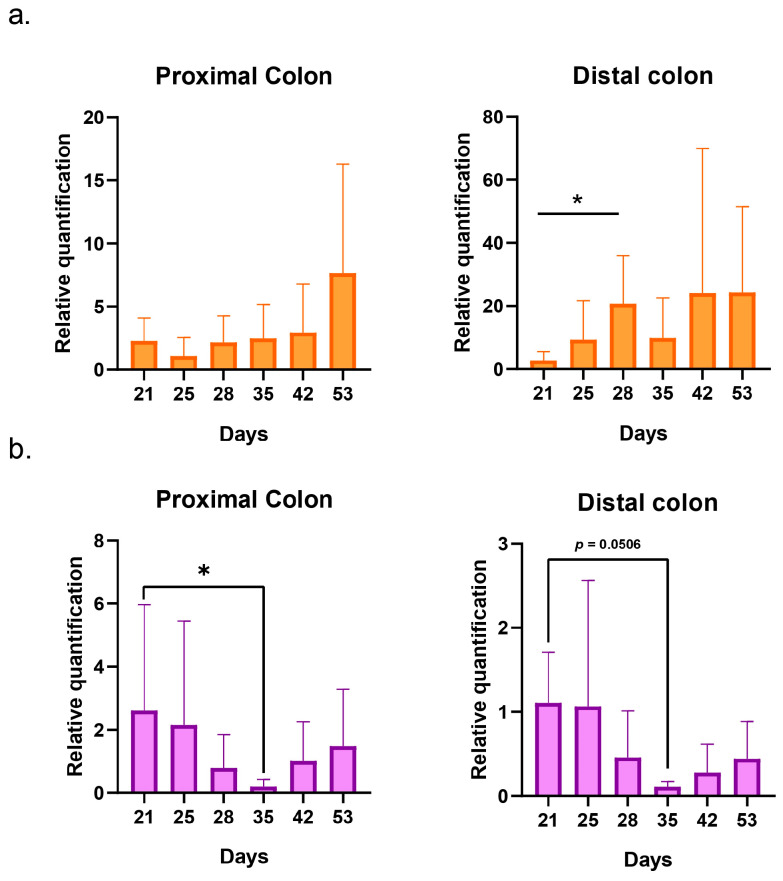
Graphical representation of the evolution of *E. coli* K1 relative quantifications in systems 2 (bacteria alone) (**a**) and 3 (bacteria + phage) (**b**) for the proximal and distal colons. The replicates represent the mean results of the three separate donors with SD. Statistical significance is indicated as *p* < 0.05 (*).

The presence of *E. coli* K1 was detectable in systems 2 and 3 from day 21, which corresponds to the first quantifiable sample after the inoculation of C5 performed in both systems. Then, the bacteria could be quantified each day until the end of the run in both systems. An increase in concentration was visible on day 28 in the distal colon of system 2 (*p* = 0.01) (Figure 3a). On day 35, a significant decrease in concentration was detected in the proximal colon of system 3. A trend of decreasing was also visible in the distal colon, although it was not statistically significant (*p* = 0.0506) (Figure 3b). The comparison of the relative quantification of systems 2 and 3 revealed no significant differences (Supplementary Materials Figure S2). Individual values of relative quantification are presented in the Supplementary Materials, Figures S3 and S4. 

#### 2.2.3. Impact on Pregnant Donors’ Microbiota

The analysis of alpha diversity showed no significant differences in the evolution of bacterial diversity over time in both proximal and distal colons of the three systems (Supplementary Materials Table S1). The Shannon diversity indexes varied between 1.18 ± 0.31 and 1.85 ± 0.2 in proximal colons and 1.92 ± 0.55 and 2.53 ± 0.39 in distal colons. The Simpson indexes varied between 0.53 ± 0.14 and 0.79 ± 0.02 in proximal colons and 0.68 ± 0.17 and 0.87 ± 0.03 in distal colons The Piélou equitability indexes varied between 0.44 ± 0.08 and 0.62 ± 0.02 in proximal colons and 0.52 ± 0.14 and 0.67 ± 0.05 in distal colons.

1. Donor 1

**Figure 4:**
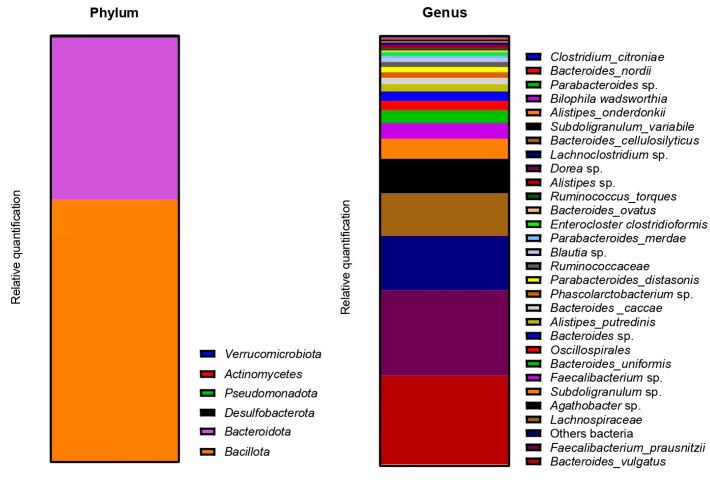
Relative abundance in bacterial composition (16S rDNA) at the phylum (**left**) and genus/species (**right**) levels in the fecal inoculum of donor 1.

The metagenetic analysis of the fecal inoculum at day 0 showed a predominance of *Bacillota* and *Bacteroidota* at the phylum level. The main genera and species represented in terms of relative abundance were *Bacteroides vulgatus*, *Faecalibacterium prausnitzii*, genera of the *Lachnospiraceae* family, *Agathobacter* sp., *Subdoligranulum* sp., *Faecalibacterium* sp., *Bacteroides uniformis*, genera of the *Oscillospirales* order, *Bacteroides* sp. and *Alistipes putredinis* (Figure 4). 

**Figure 5:**
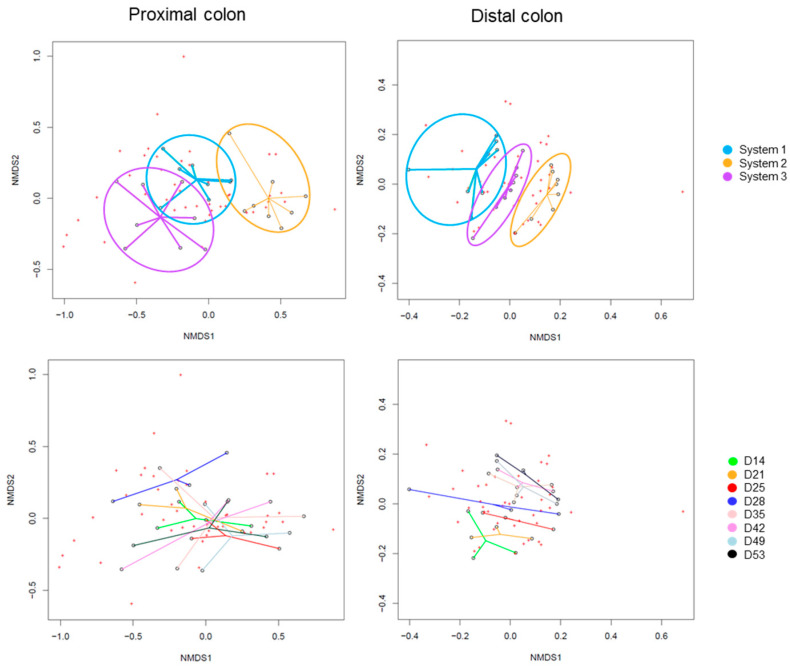
Non-Metric Multidimensional Scaling (NMDS) of Beta-diversity by systems (**up**) or by days of experiment (**down**) in proximal and distal colons of donor 1. D: day.

The Beta-diversity of all samples are represented in Figure 5 using non-metric multidimensional scaling. The HOMOVA statistical test showed no significant differences between groups, either between systems or between days of experiment. A drift of diversity (not significant) was visible over the days in the distal colon, but not in the proximal colon.

**Figure 6:**
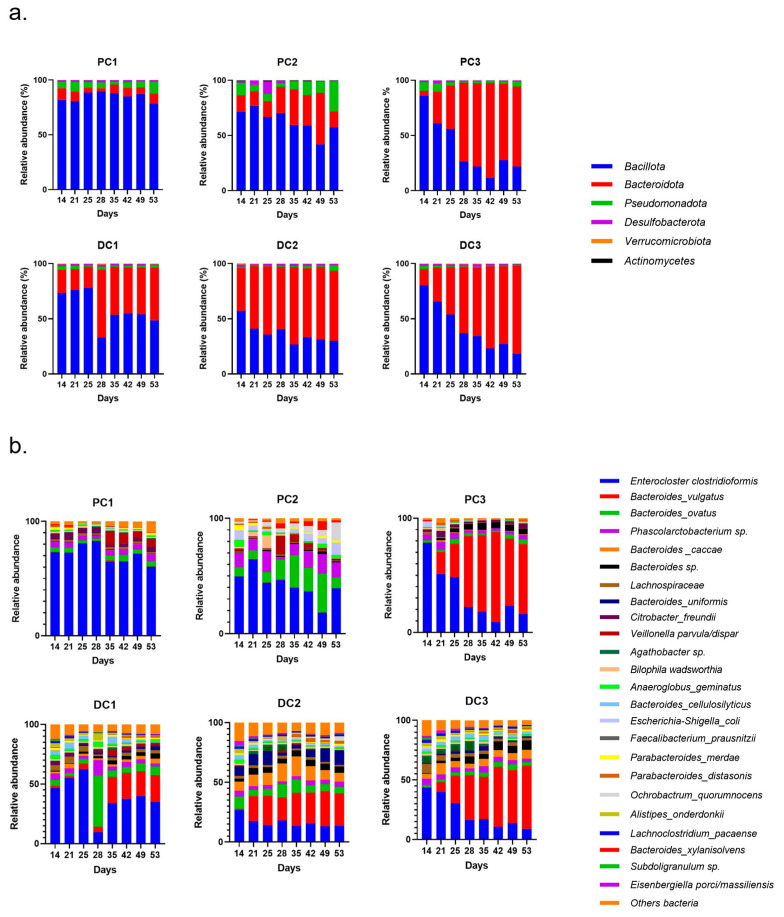
Evolution of relative abundances (16s rDNA) at the phylum (**a**) and genus/species (**b**) from day 14 (end of stabilization of the microbiota) to day 53 for donor 1. PC: proximal colon, DC: distal colon, 1: system 1, 2: system 2, 3: system 3.

The evolutions of relative abundances of phyla and genera after 14 days of stabilization are presented in Figure 6. The two main phyla were still represented by *Bacillota* and *Bacteroidota*, followed by *Pseudomonadota*. The main genera and species were *Enterocloster clostridioformis*, *Bacteroides vulgatus*, *Bacteroides ovatus*, *Phascolarctobacterium* sp. and *Bacteroides caccae*. *E. coli* was also graphically visible, mainly in the proximal colons of systems 2 and 3 (PC2 and PC3).

The monitoring of several bacterial groups by qPCR allowed variations over time to be highlighted. The timeline of the experiment, including timepoints T1, T2, T3 and T4, is described in Section 4.4. In system 1, *Bacillota* increased until T2 and then decreased at T3 in the proximal colon. In DC1, we observed an increase in *Bacillota* at T4. For *Bacteroidota*, a decrease appeared in PC1 at T2, and an increase was detected in DC1 at the end on the run. A decrease in *Gammaproteobacteria* was observed at T4 in DC1. *Actinomycetes* was decreased at T2 in both colons and at T4 in DC1. An increase in *Escherichia coli* appeared in PC1 at T3 and T4. *Akkermansia* was decreased in both colons at T3. 

In system 2, a decrease in *Bacillota* was observed in PC2 and DC2 at T4 and T3, respectively. *Bacteroidota* was first increased at T1 and then decreased at T2 in PC2. In DC2, an increase was observed at T2. A decrease in *Gammaprotebacteria* appeared in PC2 and DC2 at T3 and T2, respectively. Conversely, an increase in *Actinomycetes* was observed in PC2 and DC2 at T3 and T4, respectively. A decrease in *E. coli* in PC2 at T2 was also significant, despite the injection of C5 during this period. *Akkermansia* also showed a decrease at T2, which was visible until the end of the run. 

In system 3, a decrease in *Bacillota* appeared at T3 in the proximal colon. Several significant variations were also demonstrated in both colons for *Bacteroidota*, with an overall increase in their relative quantification. A significant decrease was observed for *Gammaproteobacteria* in PC3 at T2 and T3 and for *Actinomycetes* at T4 for both colons. The species *E. coli* showed a significant decrease in its relative quantification in PC3 at T3, and with a *p*-value = 0.055 in DC3 at T4. *Akkermansia* in the proximal colon showed a similar decrease to the one observed in PC2. The qPCR results are shown in the Supplementary Materials, Figure S5.

**Figure 7:**
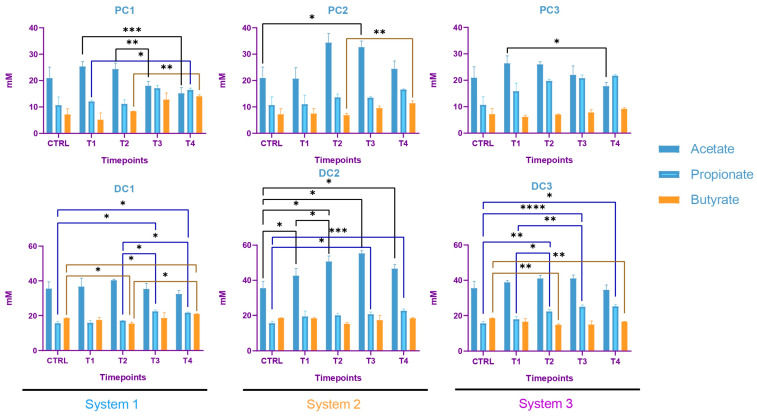
Evolution in production of short chain fatty acids (SCFA) in proximal and distal colons of the three systems of donor 1. PC: proximal colon, DC: distal colon, 1: system 1, 2: system 2, 3: system 3. Statistical significance is indicated as *p* < 0.05 (*), *p* < 0.01 (**), *p* < 0.001 (***) and *p* < 0.0001 (****).

**Figure 8:**
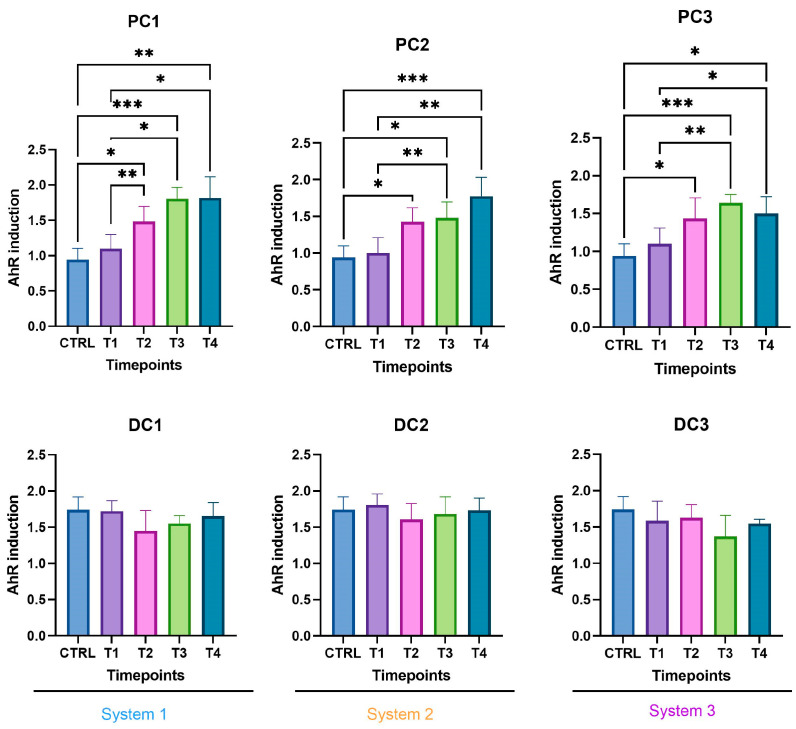
Induction of the AhR receptor in proximal and distal colons of the three systems of donor 1. PC: proximal colon, DC: distal colon, 1: system 1, 2: system 2, 3: system 3. Statistical significance is indicated as *p* < 0.05 (*), *p* < 0.01 (**), and *p* < 0.001 (***).

Some variations were observed in the SCFA concentrations and are presented in Figure 7. In system 1, a significant increase in butyrate at the end of the run and an increase in propionate at T3 and T4 were observed. Moreover, a decrease in acetate was detected in PC1 at T4. In system 2, acetate was increased in both colons, at T3 in PC2 and from T1 to T4 in DC2. An increase in butyrate and propionate appeared, respectively, in PC2 and DC2 at the end of the run. In system 3, a significant increase in propionate was visible from T2 to T4 in DC3. The same trend was visible in PC3, with no significant results (CTRL-T2: *p* = 0.0733, CTRL-T4: *p* = 0.0759). For butyrate, a decrease at T2 was followed by an increase at T4 in DC3. In PC3, a trend of increasing was observed at T4 (T2–T4: *p* = 0.09). Moreover, a significant decrease in acetate appeared at T4 in PC3. The AhR receptor was progressively and significantly induced in PC1, PC2, and PC3 and remained stable in the distal colons (Figure 8).

2. Donor 2

**Figure 9:**
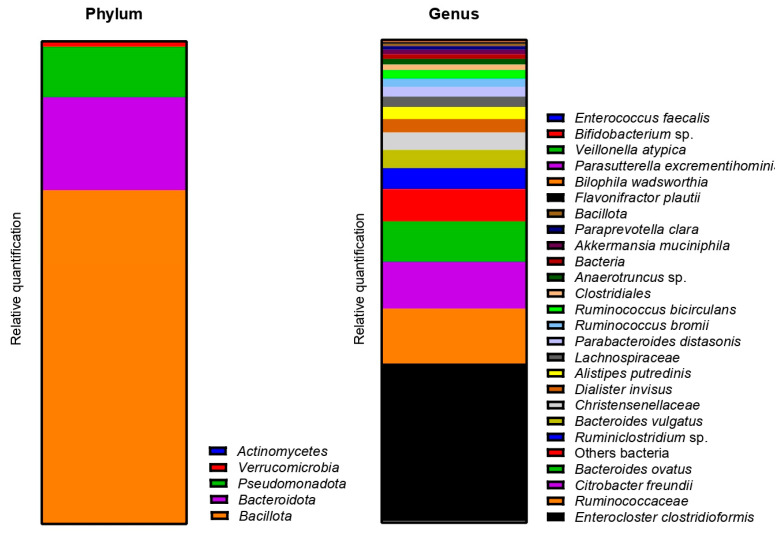
Relative abundance in bacterial composition (16S rDNA) at the phylum (**left**) and genus/species (**right**) levels, in the fecal inoculum of donor 2.

As for donor 1, the metagenetic analysis of the fecal inoculum at day 0 showed a pre-dominance of *Bacillota* and *Bacteroidota* at the phyla level. *Pseudomonadota* and *Verrucomicrobia* were also visible, as shown in Figure 9. The main genera and species represented in terms of relative abundance were *Enterocloster clostridioformis*, genera from the *Ruminococcacceae* family, *Citrobacter freundii*, *Bacteroides ovatus*, *Ruminoclostridium* sp., *Bacteroides vulgatus*, genera from the *Christensenellaceae* family, *Dialister invisus*, *Alistipes putredinis* and genera from the *Lachnospiraceae* family. 

**Figure 10:**
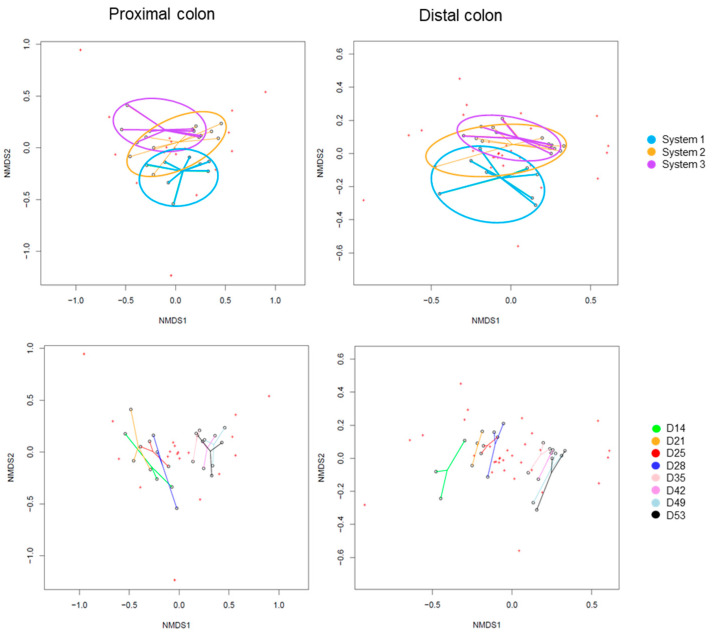
Non-Metric Multidimensional Scaling (NMDS) of Beta-diversity by systems (**up**) or by days of experiment (**down**) in proximal and distal colons of donor 2. D: day.

The Beta-diversity of all samples are represented in Figure 10 using non-metric multi-dimensional scaling. The HOMOVA statistical test showed no significant difference between groups, either between systems or between days of experiment. A drift in diversity was visible (not significant) over the days between days 14, 21, 25 and 28 and days 35, 42, 49 and 53 in both colons.

**Figure 11:**
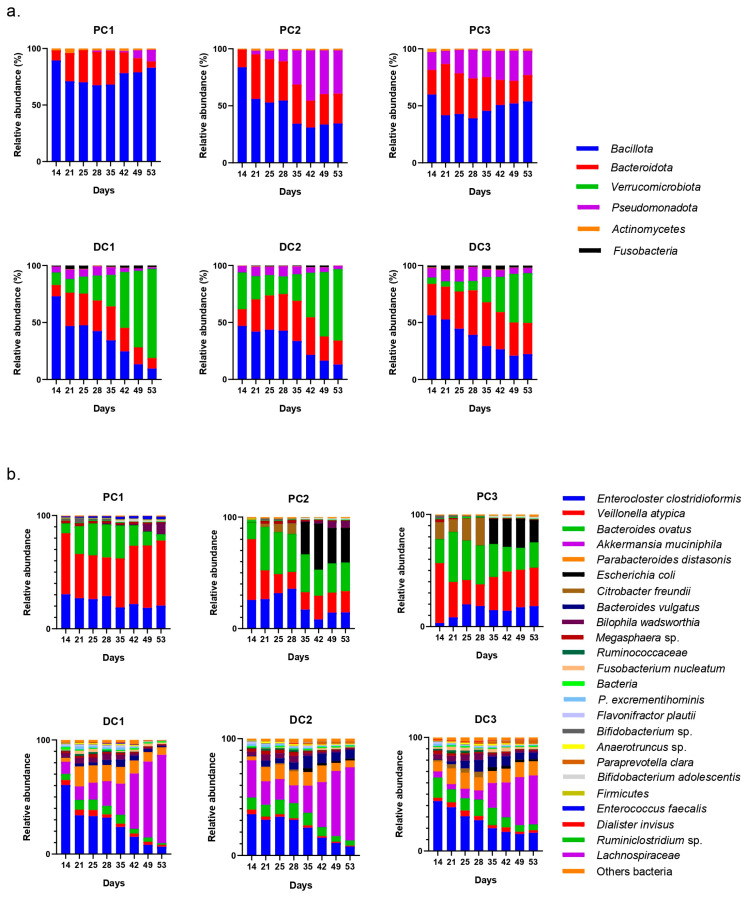
Evolution of relative abundances (16s rDNA) of phylum (**a**) and genus/species (**b**) from day 14 (end of stabilization of the microbiota) to day 53 for donor 2. PC: proximal colon, DC: distal colon, 1: system 1, 2: system 2, 3: system 3.

The evolution of relative abundances of phyla and genera after 14 days of stabili-zation are presented in Figure 11. The main represented phyla were *Bacillota*, *Bacteroidota* and *Pseudomonadota* in proximal colons. In distal colons, the third-most-represented phylum was *Verrucomicrobiota*. The main genera were *Enterocloster clostridioformis*, *Veillonella atypica*, *Bacteroides ovatus*, *Akkermansia muciniphila* and *Parabacteroides distasonis. E. coli* was also graphically visible in both PC2 and PC3.

The results of the monitoring of bacterial groups by qPCR showed some variations (Supplementary Materials Figure S6). In system 1, a significant decrease in *Bacilllota* appeared at T3 in DC1. Regarding *Gammaproteobacteria*, a decrease at T2 was followed by an increase at T3 and T4 in PC1, and decreases at T3 and T4 in DC1 were observed. A decrease in *Actinomycetes* and *Akkermansia* also appeared in PC1 at T4, and at T3 and T4, respectively. Conversely, increases in *E. coli* and *Akkermansia* were observed at T4 in DC1. 

In system 2, a significant drop in *Bacillota* and *Gammaproteobacteria* occurred at T3 in DC2. Conversely, an increase in *Gammaproteobacteria* in PC2 occurred at T4. *Actinomycetes* and *E. coli* were significantly increased at T4 in DC2. *E. coli* also increased at T3 and T4 in PC2. A variation of *Akkermansia* was observed in DC2, with a decrease at T2 followed by an increase at T4. 

In system 3, a significant decrease in *Bacillota* occurred at T4 in DC3. *Bacteroidota* were decreased at T4 in PC3. A progressive increase occurred for *Gammaproteobacteria*, from T2 to T4 in PC3. An increase occurred for *E. coli* in parallel with a decrease in *Akkermansia* at T4 in PC3. 

**Figure 12:**
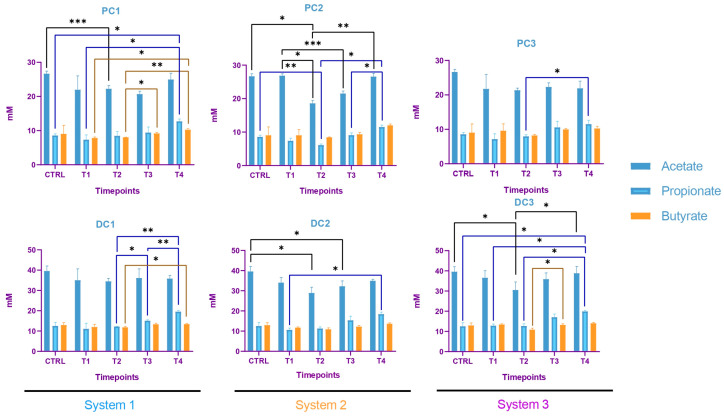
Evolution in the production of short chain fatty acids (SCFA) in proximal and distal colons of the three systems of donor 2. PC: proximal colon, DC: distal colon, 1: system 1, 2: system 2, 3: system 3. Statistical significance is indicated as *p* < 0.05 (*), *p* < 0.01 (**) and *p* < 0.001 (***).

**Figure 13:**
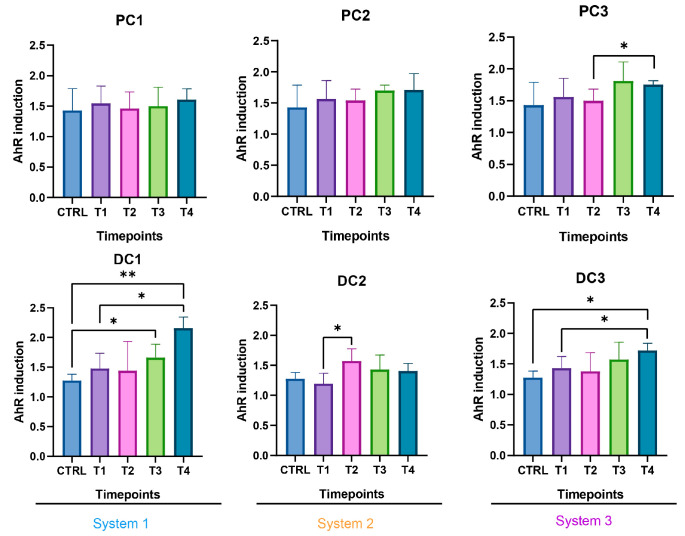
Induction of the AhR receptor in proximal and distal colons of the three systems of donor 2. PC: proximal colon, DC: distal colon, 1: system 1, 2: system 2, 3: system 3. Statistical significance is indicated as *p* < 0.05 (*) and *p* < 0.01 (**).

In system 1, increases in propionate and butyrate production appeared in both colons at the end of the run. Moreover, a decrease in acetate was detected at T2 in PC1. In system 2, acetate production dropped between T2 and T3 in both colons and then increased at T4 in PC2. The same trend was observed for propionate in PC2. In DC2, propionate increased at T4. In system 3, we also observed a decrease in acetate at T2, followed by an increase at T4 in DC3 and an increase in propionate at T4 in both colons. The SCFA productions results are presented in Figure 12. The AhR receptor was induced in DC1, with an increase at T3 and T4. An increase was also observed at T2 in DC2 and in both PC3 and DC3 at T4. (Figure 13).

3. Donor 3

**Figure 14:**
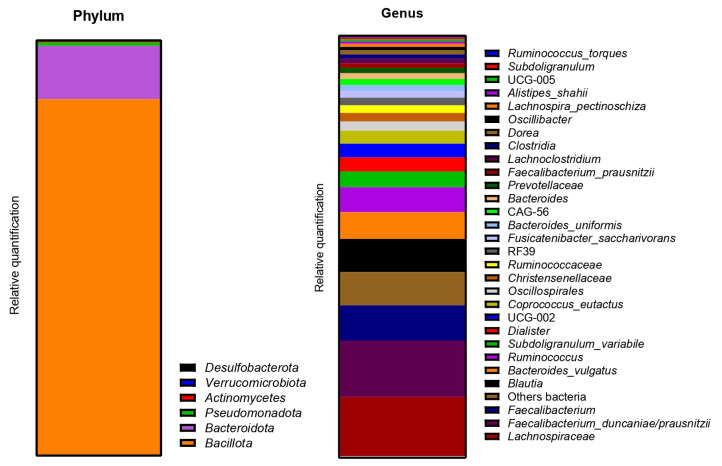
Relative abundance in bacterial composition (16S rDNA) at the phylum (**left**) and genus/species (**right**) levels in the fecal inoculum of donor 3.

The metagenetic analysis of the fecal inoculum at day 0 showed a predominance of *Bacillota* and *Bacteroidota* at the phyla level (Figure 14). The main genera/species represented in terms of relative abundance were genera and species from the *Lachnospiraceae* family, *Faecalibacterium*, *Blautia*, *Bacteroides vulgatus*, *Ruminococcus*, *Subdoligranulum variabile*, *Dialister*, *Coprococcus eutactus*, genera from the *Oscillospirales* order and genera from the *Christensenellaceae* family. 

**Figure 15:**
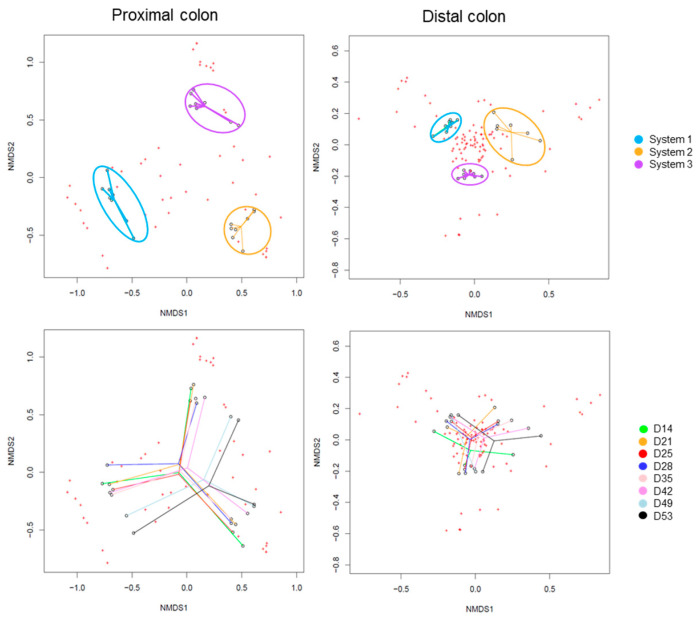
Non-Metric Multidimensional Scaling (NMDS) of Beta-diversity by systems (**up**) or by days of experiment (**down**) in proximal and distal colons of donor 3. D: day.

The Beta-diversity of all samples is represented in Figure 15, using non-metric multi-dimensional scaling. The HOMOVA statistical test showed no significant differences between groups, either between systems or between days of experiment. A clustering was visible by system in both colons. A drift in diversity (not significant) was also visible at days 49 and 53 in the proximal colon.

**Figure 16:**
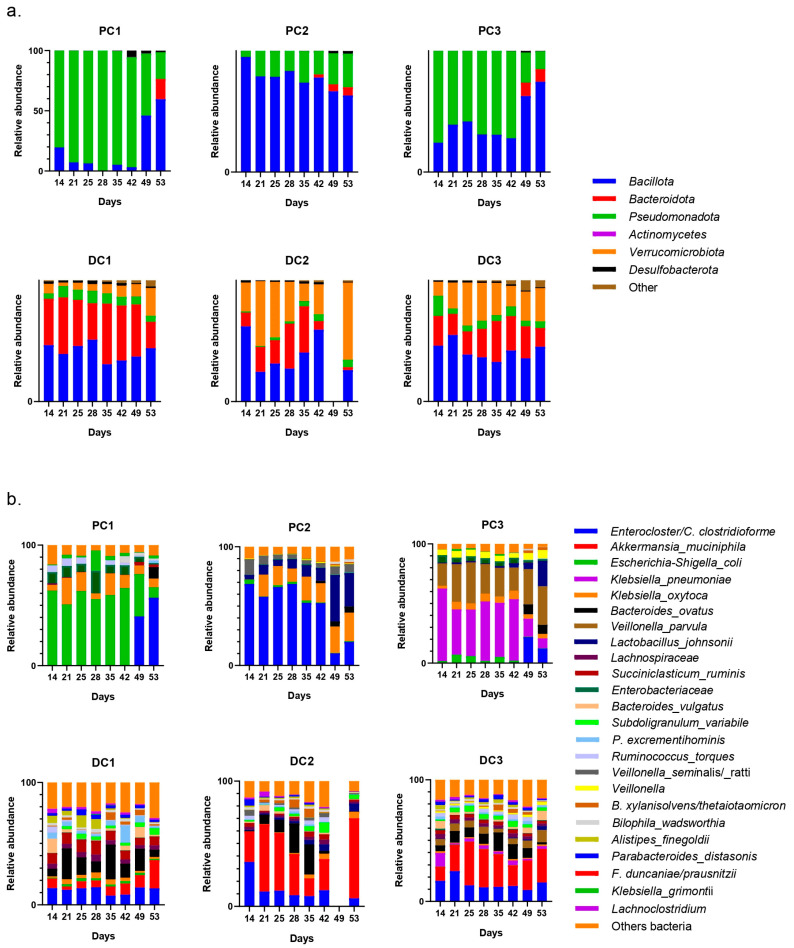
Evolution of relative abundances (16s rDNA) of phylum (**a**) and genus/species (**b**) after 14 days of stabilization of the microbiota from donor 3. PC: proximal colon, DC: distal colon, 1: system 1, 2: system 2, 3: system 3.

After the stabilization period, the bacterial composition of the microbiota of the proximal colon stabilized differently in each system (system 1, 2 and 3), despite the fact that no anomalies in pH, temperature, or tightness were detected during this period. This was mainly visible at the genus level, with a predominance of *E. coli* in PC1, *Enterocloster clostridioformis* in PC2 and *Klebsiella pneumoniae* in PC3 with, as a repercussion, an important representation of *Pseudomonadota* in PC1 and PC3 at the phyla level. Despite these data, the results were analyzed by separating the systems and colons as performed for donors 1 and 2. It is important to keep in mind, however, that it was not possible to determine whether one or none of the systems were able to represent the bacterial diversity present in the starting inoculum. The evolution of the relative abundance is shown in Figure 16. 

Some variations in the relative quantifications by qPCR were observed and are shown in Figure S7. In system 1, increases in *Bacillota* and *Gammaproteobacteria* appeared at T4 in PC1. In DC1, a decrease was detected in *Gammaproteobacteria* and *Akkermansia* at T3. An increase in *Actinomycetes* was demonstrated at T3 and T4 in PC1 and DC1, respectively, and a decrease at T4 for PC1. For *E. coli*, a significant increase appeared at T2 in DC1. 

For system 2, increases were detected in PC2 for *Bacillota* at T2 and *Bacteroidota* at T4. In DC2, significant increases in *Gammaproteobacteria*, *Actinomycetes* and *Akkermansia* were observed at T4. In system 3, an increase in *Bacteroidota* at T4 was detected in PC3. No other significant changes were highlighted in system 3.

**Figure 17:**
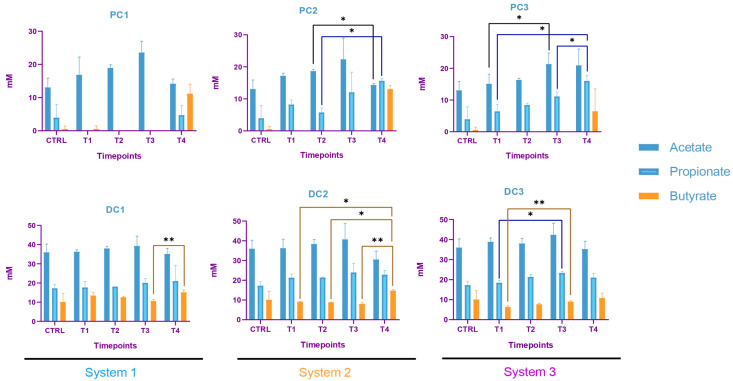
Evolution in production of short chain fatty acids (SCFA) in proximal and distal colons of the three systems of donor 3. PC: proximal colon, DC: distal colon, 1: system 1, 2: system 2, 3: system 3. Statistical significance is indicated as *p* < 0.05 (*) and *p* < 0.01 (**).

**Figure 18:**
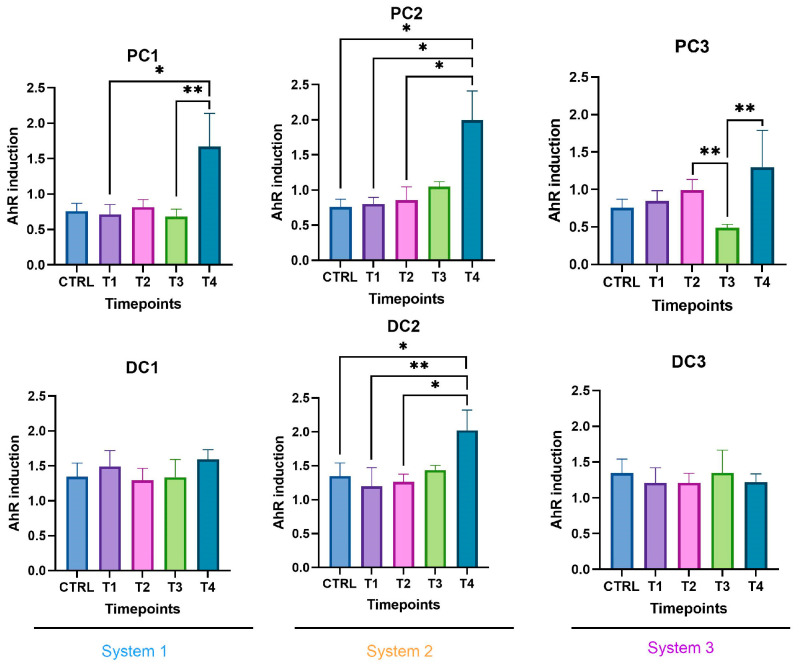
Induction of the AhR receptor in proximal and distal colons of the three systems of donor 3. PC: proximal colon, DC: distal colon, 1: system 1, 2: system 2, 3: system 3. Statistical significance is indicated as *p* < 0.05 (*) and *p* < 0.01 (**).

The SCFA production in system 1 remained stable during the run, except for an increase in butyrate in DC1 at T4. In system 2, a decrease in acetate and an increase in proprionate were observed at T4 in PC2. In DC2, an increase in butyrate appeared at the end of the run. In system 3, acetate and propionate were increased at T3 and T4, respectively, in PC3. In DC3, propionate and butyrate were both increased at T3 (Figure 17). The AhR was more strongly induced at T4 for PC1, both PC2 and DC2, and PC3, despite a drop at T3 in PC3 (Figure 18).

## 3. Discussion

In this study, the aim was to evaluate the therapeutic potential of K1_ULINTec4 in the elimination of an *E. coli* K1 strain in the microbiota of pregnant women to prevent its transmission and the development of sepsis and meningitis in newborns. This preventive treatment would not be enough to completely eliminate the risk of developing an infection in the newborn. Indeed, these strains can come from other sources, such as the environment, equipment, and hospital staff [36,37]. Targeting these bacteria in the intestinal microbiota of pregnant woman could nevertheless reduce the risk of infection. The aim of this study was to evaluate not only the efficacy of phage therapy, but also its impact on the bacterial populations of the intestinal microbiota.

The first objective was to select an appropriate phage concentration to be inoculated into the SHIME^®^ model. Different concentrations, expressed as multiplicity of infection (MOI), had been previously tested in an in vivo *G. mellonella* model [33]. In this study, this experiment was repeated with the C5 strain and three different MOI (100, 10 and 1) of K1_ULINTec4. The treatment increased the survival rate of the larvae independently of the tested concentrations. Therefore, an MOI of 1 was chosen for the experiments conducted in the gastrointestinal model. This ratio corresponds to a concentration of 10^10^ PFU/administration in the SHIME model, corresponding to 10^8^ PFU/mL after distribution in the volume of the bioreactors, which is consistent with the doses generally used therapeutically in humans [38]. Moreover, this dose was the highest achievable dose under the amplification and concentration conditions used in this study.

The K1_ULINTec4 phage was found to be able to persist in the SHIME^®^ model, as evidenced by its persistence being similar to the theoretical persistence of the inert molecule in the proximal colon. This aptitude is not commonly seen, as described in other studies where phages tend to disappear more rapidly [29,30,39]. In most cases, this is certainly due to the pH conditions experienced and/or the action of bile and pancreatin [39]. For donors 1 and 3, the fact that the phage was eventually washed out of the system was also a sign that K1_ULINTec4 could not find a host to support its replication in the commensal bacteria of the intestinal microbiota. However, a low quantifiable phage titer was detected at the end of the run in the distal colon of donor 2. It is therefore possible that the phage replicated in this system in the absence of *E coli* K1 inoculation. This could be due to the presence of low levels of *E coli* K1 in the donor’s intestinal microbiota not being detected either by qPCR screening or by concentration monitoring during the run. In parallel, it was observed that when both C5 and K1_ULINTec4 (system 3) were inoculated, the phage concentration differed significantly from system 1 eighteen days after the last phage inoculation in both colons, meaning that K1_ULINTec4 was able to replicate using the C5 strain in the model. Its concentration even tended to stabilize, highlighting the possibility that the phage continues to replicate in the presence of its host bacteria without a complete elimination.

Regarding *E. coli* K1 inoculated in system 2 (C5 alone) and 3 (C5 + K1_ULINTec4), it could be detected from the first sampling day until the last day of the run, reflecting the persistence of the bacteria in the system even after the inoculations were stopped. Unfortunately, despite the effective replication of the phage in system 3, the phage treatment did not completely eliminate the bacterial load. A significant decrease and a decreasing trend occurred at T3, respectively, in PC3 (*p* = 0.0179) and DC3 (*p* = 0.0506), which may reflect a transient action of the phage. The coexistence of both phages and bacteria in system 3 may indicate the appearance of resistant clones. One possible explanation is that *E. coli* K1 identified by qPCR may have ceased production of their K1 capsule, resulting in a decreased or inhibited phage replication. This may result in a reduction in the virulence of the bacteria that were inoculated. This phenomenon has previously been observed by other researchers, who have demonstrated that phages able to selectively degrade the PSA capsule on the surface of *E. coli* K1 using endosialidases can alter its phenotype, reduce bacteremia, and decrease mortality rates in rats [28]. The phage K1_ULINTec4 encodes an endosialidase, allowing it to target the K1 capsule of *E. coli* [33]. It would be interesting to evaluate the virulence of *E. coli* K1 isolates after contact with the phage. Indeed, the resistance mechanisms that bacteria deploy against phages generally lead to fitness trade-offs that can reduce antibiotic resistance, virulence or colonization abilities [40]. Nevertheless, the phenomenon of pleiotropy can also generate trade-up effects, especially demonstrated in terms of antibiotic resistance [41,42]. It is therefore important to study these effects when evaluating phage-based treatments.

In order to reflect diversity in the Triple-SHIME experiments, three different pregnant donors were selected. However, the presence of donor inter-variability did not allow for an analysis of the metagenetic results together. Therefore, each donor was analyzed separately. To be able to go further into the statistical interpretation of the results, it would have been interesting to carry out technical replicates of each donor, which could not be performed in this study. Nevertheless, the different analyses carried out in the study are complementary and help to reinforce the obtained results.

The metagenetic analysis of the donor 1 fecal inoculum led to its classification as enterotype 1 due to the abundance of the genus *Bacteroides* [43,44]. After the stabilization of the intestinal microbiota in the model, the main genera detected corresponded to those of the starting inoculum, but with a different level of abundance. Indeed, the conditions of the model favored the development of some species to the detriment of others in each part of the colon. For example, the acidic pH of the proximal colons and the presence of highly fermentable nutrients was more favorable to the development of bacteria of the phylum *Pseudomonadota*. However, some differences are to be noted, such as the important abundance of bacteria from the genus *Enterocloster clostridioformis* (formerly *Clostridium clostridioforme* and *Lachnoclostridium clostridioforme*), an acetate and propionate producer and member of the family *Lachnospiracae*, frequently found in abundance in the intestinal microbiota [45]. When comparing the results of the monitoring of certain bacterial groups by qPCR with the results of metagenetics, certain concordances were observed: a decrease in the abundance of *Bacillota* in PC2, DC2 and PC3 at the end of the run, and an increase in *Bacteroidota* in DC1, PC2 and DC2, PC3 and DC3. A decrease in *Gammaproteobacteria* in PC3 and of *E. coli*, belonging to this class, in PC3 and DC3 seems to indicate an action of the phage on the inoculated bacteria. A decrease in *E. coli* was also visible in PC2, but was not maintained over time. The gut microbiota’s bacterial composition has a significant impact on short-chain fatty acids (SCFAs) production in the colon. SCFAs are the result of the fermentation of non-digestible dietary fibers by colonic bacteria, and different bacterial species have specific preferences for fermentation substrates and therefore produce different SCFAs in varying amounts [46]. In distal colons, the significant increase in propionate from T3 is consistent with the qPCR increase in the *Bacteroidota* phylum and is represented by a greater abundance of *Bacteroides ovatus* and *Bacteroides vulgatus*. In this study, the AhR activity can be linked to SCFAs productions. Indeed, previous studies have shown that butyrate, propionate and acetate can trigger AhR-responsive gene expression in vitro in a dose-dependent manner, with a greater sensitivity of butyrate (from 0.5 mM), followed by propionate (from 1 mM) and acetate (from 20 mM) [47,48]. SCFAs, particularly butyrate, can increase AhR activity without directly binding to the receptor. Butyrate acts as a histone deacetylase HDAC inhibitor (iHDAC), which results in increased AhR recruitment in the presence of agonist tryptophan-derived metabolites [49]. Increases in butyrate and propionate in PC1, butyrate in PC2 and propionate in PC3 resulted in significant AhR activity in proximal colons. 

The predominance of *Bacillota*, including *Ruminococcus*, classified donor 2 as enterotype 3 [43,44]. After stabilization, the predominant species present in the starting inoculum, *Enterocloster clostridioformis* and *Bacteroides ovatus*, were maintained. The abundance of *Veillonella atypica* and *Akkermansia municiphila* became more prominent in the proximal colon for *Veillonella*, and in both colons for both species. A drop in *Bacteroidota* was visible at the end of the run, using both qPCR and metagenetic analyses, in PC2 and PC3. An increase in the *Gammaproteobacteria* class was detected by qPCR in the three proximal colons, and followed the same trend in the relative abundances of *Pseudomonadota*. This significant increase could be explained by the increase in *Bilophila wadsoworthia* species in PC1, while it would rather reflect an increase in *E. coli* in PC2 and PC3, as supported by the corresponding qPCR results. A decrease in *Bacillota* appeared in the three distal colons at the end of the run. In parallel, the genus *Akkermansia* was significantly decreased in PC1 and PC3 and increased in DC1 and DC2. Regarding volatile fatty acid production, a decrease in acetate concentration occurred in PC1, PC2, DC2 and DC3 at T2 and then increased again until T4. At the end of the run, increases in propionate were visible in all systems, and the butyrate increased in system 1 and DC3. The activation of the AhR receptor was found to be significant in PC3 and appeared to correlate with propionate production. In DC1 and DC3, the activity of the AhR receptor was increased at the end of the run, as for propionate and butyrate productions. 

The gut microbiota of donor 3 was classified as enterotype 3, as for donor 2 [43,44]. Very surprisingly, after the stabilization of the microbiota in the model, the composition of the bacterial populations differed between the three systems. This was mainly visible in the metagenetic results of the proximal colons. Only a few predominant taxa persisted in visible abundance in the model: *Lachnospiracae*, *Bacteroides vulgatus*, *Subdoligranulum*, *Ruminococcus torques* and *Lachnoclostridium*. Interestingly, the major taxa that persisted after stabilization were the same as the predominant species found in donor 2, *Enterocloster clostridioformis*, *Bacteroides ovatus*, *Veillonella* and *Akkermansia municiphila*, found in low abundance in the starting inoculum. This reflected a bias in the model that reinforced certain genera more adapted to the applied conditions. No technical problems were detected during the stabilization phase, so these differences are difficult to explain. One hypothesis could be undetected variations induced by the model during the stabilization phase. Referring to donor 2, and taking into account the similarity at the enterotype level, system 2 seemed to have stabilized in the most appropriate way. Few variations were highlighted in qPCR, compared to other donors. The *Bacteroidota* phylum, composed of the main propionate-producing species, was absent from the proximal colons throughout the experiment, except at the end of the run [50]. This observation was confirmed by the metagenetics results, but also by the SCFA production. In PC1, the propionate concentration, low at the start (4 mM), became undetectable until T4, when production was observed again. This observation differs from PC2 and PC3, where a production of propionate was found in the absence of *Bacteroidota* until T3. The production of propionate in that case could be explained by the presence of *Veillonella* in both PC2 and PC3, and not in PC1 [51]. The evolution of the butyrate concentration followed the same trend as the propionate in PC1, with a low concentration at the start followed by an absence of production and an increase at the end. This was observed in the three proximal colons. These results were consistent with the levels of AhR activation, and were correlated with the genus *Bacteroides ovatus*, which appeared at the end of the run in the three bioreactors [52].

All the results related to the intestinal microbiota showed that the effects observed were donor dependent. This was expected, given the inter-variability between donors. Working with three different donors made it possible to evaluate the treatment, taking into account the inter-individual variability, which would not be possible for a single inoculum repeated three times, or an inoculum composed of a mixture of several donors. Significant variations were observed in the different systems, but did not make it possible to demonstrate a common and significant effect of the three applied treatments (phage alone, bacteria and bacteria + phage) in the systems. In addition, most of the significant results were observed at the end of the run, which can be explained by variations due to the long duration of the experiments. These observations confirm a limited and donor-dependent impact of the phage and *E. coli* K1 on the intestinal microbiota. In addition, Alpha- and Beta-diversity analyses of the three donors did not reveal any significant differences, once again demonstrating the limited impact of these treatments. However, the K1_ULINTec4 phage inoculated into the model in the presence of *E. coli* K1 resulted in a decrease in bacterial concentration after the cessation of injections, and a prolonged persistence of the phage compared to its persistence when inoculated alone. These observations help to confirm that K1_ULINTec4 replicated in the model using the inoculated *E. coli* K1. Additionally, the possible appearance of phage-resistant mutants may have allowed *E. coli* K1 to persist in the model. The genomic analysis and evaluation of these *E. coli* K1 mutants resistant to phage K1_ULINTec4 could provide further insight into the pathogenicity of these strains.

## 4. Materials and Methods

### 4.1. Strains and Culture

The neonatal meningitis *E. coli* (NMEC) strain C5 “Bort” (O18ac:K1:H7) was used throughout the study. It was originally isolated in 1975 from cerebrospinal fluid of a newborn [53]. It was purchased from the American-type culture collection (ATCC) (LGC standards, Molsheim, France) and is referred to as *Escherichia coli* (Migula) Castellani and Chalmers ATCC 700973. Cultures were grown in LB Lennox broth at 37 °C. The phage vB_EcoP_K1_ULINTec4 was isolated in 2020 in Liège from wastewater using O18:K1 avian pathogenic *E. coli* strains (APEC45). This K1-dependent phage, belonging to the *Vectrevirus* genus, was characterized in vitro and in vivo using the *Galleria mellonella* model [33]. In this study, cesium chloride phage purification was performed for the determination of the multiplicity of infection (MOI) in *G. mellonella*, as described previously (Antoine et al. [33]). Phage cultures used in the SHIME^®^ model were grown following a previously described propagation protocol, with some modifications [33]. After filtration (0.22 µm), phage lysate was concentrated by ultracentrifugation at 50,000× *g* for 3 h. The pellet was resuspended in phosphate-buffered saline solution (PBS) and ultracentrifuged a second time using the same parameters. Finally, the pellet was resuspended in PBS and stored at 4 °C before use. Phage titrations were performed using serial dilutions and a top agar overlay method [33].

### 4.2. Determination of the Optimal Phage Concentration in the Galleria mellonella Model

The preliminary and main experiments were performed as described previously (Antoine et al. [33]). To the determine optimal bacterial inoculation dose, 6 groups of 10 larvae were inoculated with C5 at different concentrations, ranging from 10² CFU/10 µL to 10^6^ CFU/10 µL or PBS. The optimal inoculation dose was expected to result in 90–100% lethality after 4 days.

In the main experiment, 180 larvae were divided in 6 groups (Table 1). The larvae were injected twice at 1 h intervals, and the survival rate was assessed every 24 h for 96 h. 

### 4.3. Fecal Material Collection and Screening

The collection of the human fecal material and its research use was approved by the Ethical Committee of the Liège University Hospital (ULiège, Liège, Belgium; file number 2022/274). The three adult donors were, respectively, 30, 35 and 36 years old and met the following criteria: being a healthy pregnant woman between 18 and 40 years old, not following any particular diet (e.g., vegetarian, vegan, high protein, etc.), being antibiotic-free for at least 3 months before feces collection, not presenting any medical conditions, such as diabetes or gastrointestinal diseases.

Fecal samples were stored anaerobically at 4 °C before being processed as described previously (Goya-Jorge et al. [47]). They were stored at −80 °C until their use for experimentations.

A screening for naturally occurring *E. coli* K1 in the fecal samples from the donors was performed using qPCR targeting the *neuB* gene [54]. DNA extraction was performed using the QIAamp PowerFecal Pro DNA Kit (Qiagen, Hilden, Germany), following the manufacturer instructions. Real-time PCR was run using CFX96 Touch Real-Time PCR Detection System (Biorad, Hercules, CA, USA), according to the manufacturer’s recommendations, in 20 µL reaction volume using the FastGene^®^ Probe 2 × qPCR Universal Mix (Nippon genetics, Tokyo, Japan). Details about primers, probes and cycle steps are described by Antoine et al. [33]. Samples were run in triplicate, including positive, negative and no template (NTC) controls.

The presence of phages from feces that could potentially infect the C5 strain was also assessed using an enrichment method [55]. Briefly, 1 g of feces was diluted 10 times in PBS and then filter-sterilized using a 0.22 µm sterile syringe filter (ref.514-0073, VWR, Leicestershire, UK). Then, the filtrate was added to an equivalent volume of twice-concentrated LB Lennox (1 mM CaCl_2_, 1 mM MgSO_4_, Sigma-Aldrich, Saint-Louis, MO, USA), as well as 100 µL of C5 culture at an optical density (OD) of 0.25. A sterility control and a growth control were also included. All tubes were incubated at 37 °C until potential lysis occurred. Samples were then centrifuged and filtered-sterilized (0.22 µm) before spreading on an LB Lennox agar plate and covered with a bacterial overlay (OD: 0.25). Plates were incubated at 37 °C for 24h to check the presence of plaques of lysis.

### 4.4. Human Gastrointestinal Model Set-Up and Experimental Design

**Figure 19:**
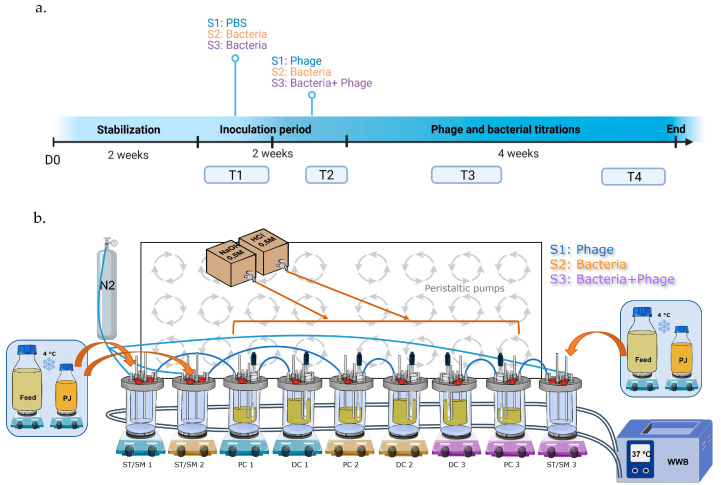
Timeline of experiment (**a**) and experimental setting of the Triple-SHIME (**b**). S1: system 1, S2: system 2, S3: system 3, D0: day 0 T1: timepoint 1 (days 14 and 21), T2: timepoint 2 (days 25 and 28), T3: timepoint 3 (days 35 and 42), T4: timepoint 4 (days 49 and 53), ST/SM: stomach/small intestine, PC: proximal colon, DC distal colon, PJ: pancreatic juice, N2: nitrogen, WWB: warm water bath.

Three independent triple-SHIME^®^ runs were performed to reproduce the colon microbiota of the three different donors, using standard conditions, as previously described [56]. Each run was composed of three independent experiments conducted in three separate dynamic systems, working in parallel. Each system was composed of bioreactors representing the stomach/small intestine (ST/SM), the proximal colon (PC) and the distal colon (DC). The temperature was maintained at 37 °C using a continuous warm water flow between the double glass wall of the bioreactors. Anaerobic conditions were maintained throughout the experiment and the headspace was flushed once a day with a nitrogen flow (2.0 L/min). The pH of the colon bioreactors (PC: 5.6–5.9, DC: 6.6–6.9) was recorded with internal pH probes and regulated with the automatic addition of acid (HCl 0.5M) or base (NaOH 0.5M) solutions (ChemLab, Zedelgem, Belgium). Verifications of the pH range were performed three times a week during the entire experiment with an external pH measurement (Mettler-Toledo, Zaventem, Belgium). On the inoculation day, PC and DC bioreactors were filled with 500 mL and 800 mL of nutritional medium, respectively (ref: PD-NM001B, Prodigest, Ghent, Belgium), referred to as feed. Then, defrosted feces were added to the bioreactors at proportional volumes (PC: 25 mL, DC: 40 mL). The 3 systems were continuously maintained under agitation (300 rpm) and automatically fed three times a day with 140 mL of feed and 60 mL of a medium simulating bile and pancreatic juice (PJ) (NaHCO_3_-pancreatin-bile salts, Prodigest, Ghent, Belgium). The experimental set-up is illustrated in Figure 19b.

Each run was conducted for 8 weeks. After feces inoculation in the 3 systems, the microbiota was stabilized for 2 weeks [57]. A 10^8^ CFU/mL concentration was chosen based on a previous study assessing the colonization of *E. coli* K1 in a mouse gastrointestinal tract [21]. System 1 was inoculated with K1_ULINTec4 only, system 2 with C5 only and system 3 with both C5 and K1_ULINTec4. Each inoculation had a volume of 10 mL of either PBS resuspended culture of C5 at a concentration of 10^10^ CFU/mL PBS or K1_ULINTec4 at a concentration of 10^10^ PFU/mL. Bacteria, phage and PBS were directly injected into the bioreactors using a 10 mL syringe with needle. After stabilization, system 1 was inoculated with PBS, while systems 2 and 3 were inoculated with C5 on a daily basis for one week. Then, system 1 was inoculated with both PBS and K1_ULINTec4, system 2 with both C5 and PBS and system 3 with both C5 and K1_ULINTec4 at one hour intervals, respectively. All the systems continued to run for 4 more weeks. Samples in colon bioreactors were collected three times a week and stored at −20 °C until extraction and analysis. Additional 2 mL samples were collected to perform phage titrations by culture. A timeline summarizing the main steps of the run is shown in Figure 19a.

The persistence of K1_ULINTec4 in systems 1 and 3 was compared to the theoretical persistence in the system, which was calculated taking into account the transfer and removal of fluids [29,30].

### 4.5. 16S rDNA Gene Sequencing and qPCR of Selected Bacterial Groups

The DNA was extracted and sequenced using the same pipeline described in Laforêt et al. [29]. The selected timepoints were chosen to cover the most important periods of the run (T1: D14-D21; T2: D25-D28; T3: D35-D42, T4: D49-D53). They are represented in the timeline of Figure 19. Raw metagenetics data are available in the Supplementary Material.

Seven taxa were targeted by qPCR to follow specific bacterial groups: *Bacillota* (formerly *Firmicutes*), *Bacteroidota* (formerly *Bacteroidetes*) and *Actinomycetes* (formerly *Actinobacteria*) as 3 of the main prevalent phyla of the intestinal microbiota, *Gammaproteobacteria* [58], *E. coli* [59] and *E. coli* K1 [54] to follow the impact of phage and *Akkermansia municiphila* [60] as a gut health marker. Indeed, *Akkermansia municiphila* is known as a probiotic for its beneficial effects on health, and particularly its action on metabolic diseases, inflammatory bowel diseases, diseases of the central nervous system and many others [61,62]. Real-time PCR assays were performed using a CFX96 Touch Real-Time PCR Detection System (Biorad, Hercules, CA, USA), in 96-well plates (Nippon Genetics Europe, Düren, Germany) in technical duplicates, in 20 µL reaction volume. Except for *E. coli* K1 (see protocol in Section 4.3), the enzyme used was the Takyon™ No ROX SYBR MasterMix, and the primers and probe (Eurogentec, Seraing, Belgium) were used at a final concentration between 300 nM and 500 nm, respectively. The following steps were applied: initial denaturation at 95 °C for 5 min, followed by 35 cycles of denaturation at 95 °C for 15 s, annealing at a specific temperature depending on the target (Supplementary Materials Table S1) for 15 s and elongation at 72 °C for 30 s followed by a final elongation at 72 °C for 5 min. Then, melt-curves (65–95 °C) were performed to assess the specificity of the assay. A relative quantification using the 2^−ΔΔCt^ method was performed to standardize the results for the total bacterial population [63].

### 4.6. Short-Chain Fatty Acids Analysis

Collected samples were centrifuged at 17,000× *g* for 5 min at room temperature. The supernatants were filtered-sterilized at 0.22 µm using syringe filters (ref.514-0073, VWR, Leicestershire, UK) to remove *E. coli* K1 before analysis. Solid-phase microextraction (SPME) followed by gas chromatography coupled to mass spectrometry (GC–MS) was used to quantify the short-chain fatty acids (SCFA), following the protocol developed by Douny et al. [64] and described in Laforêt et al. [29]. 

### 4.7. AhR Activity and Cytotoxicity Assays

AhR activity assay was conducted as described previously by Goya-Jorge et al. [47]. To evaluate the activation of the AhR receptor, AhR_HT29-Lucia (Invivogen, Toulouse, France), a human colon adenocarcinoma AhR-reporter cell line, was used. Cells were cultured according to the manufacturer’s recommendations, in DMEM supplemented with 4.5 g/L glucose, 2 mM L-glutamine, 10% of FBS and antibiotics: 100 μg/mL Pen/Strep, 100 μg/mL of Normocin and 100 μg/mL of Zeocin. They were incubated in 75 cm^2^ culture flasks at 37 °C in 5% CO_2_. Passages were performed when a confluence of 80–90% was reached. Cells were washed with PBS and detached using a 0.25% solution of trypsin-EDTA before seeding them in new flasks. For the experiment, AhR_HT29 Lucia cells were seeded at a concentration of 3.0 × 10^5^ cells/mL in CellStart® 96-well microplates and incubated overnight before treatment. After 24 h of exposure to the supernatants of the bioreactor samples, 20 μL of the cell supernatant was transferred to Nunc ™ white 96-well plates before adding 50 μL/well of Quanti-Luc™ assay reagent. Bioluminescence was measured using a luminometer (ORION II, Berthold Detection System, Pforzheim, Germany). In parallel, cell viability was assessed using the 3-(4,5-dimethylthiazol-2-yl)-2,5-diphenyltetrazolium bromide (MTT) bioassay [65,66]. The MTT formazan absorbance was read at 550/630 nm using an ELX800TM microplate reader spectrophotometer (Agilent BioTek Inc., Winooski, VT, USA). All assays were performed in technical triplicate.

### 4.8. Statistical Analysis

Diversity indexes were calculated using R with Rcmdr and vegan packages. All other statistical analyses and graphical representations were performed using GraphPad Prism version 8.0.2 for Windows, GraphPad Software (San Diego, CA, USA). After assessing the normality of the results, a repeated measures ANOVA was performed paired with Tukey’s multiple comparison test. A Friedman test paired with Dunn’s multiple comparison test was used when the results were not normal.

## Figures and Tables

**Table 1:** Groups of larvae used in the survival experiment.

	Groups	1st Injection	2nd Injection
1	C5 + ULINTec4 MOI 100	C5: 10^6^ CFU/10 µL	K1_ULINTec4: 10^8^ PFU/10 µL
2	C5 + ULINTec4 MOI 10	C5: 10^6^ CFU/10 µL	K1_ULINTec4: 10^7^ PFU/10 µL
3	C5 + ULINTec4 MOI 1	C5: 10^6^ CFU/10 µL	K1_ULINTec4: 10^6^ PFU/10 µL
4	C5 + PBS	C5: 10^6^ CFU/10 µL	PBS: 10 µL
5	PBS + ULINTec4 MOI 100	PBS: 10 µL	K1_ULINTec4: 10^8^ PFU/10 µL
6	PBS + PBS	PBS: 10 µL	PBS: 10 µL

PBS: phosphate-buffer saline, CFU: colony-forming unit, PFU: plaque-forming unit.

## Data Availability

Not applicable.

## Supplementary Materials

The following supporting information can be downloaded at: https://www.mdpi.com/article/10.3390/ijms241310580/s1.
