# Supplementary material for: Phage Targeting Neonatal Meningitis E. coli K1 In Vitro in the Intestinal Microbiota of Pregnant Donors and Impact on Bacterial Populations
Source: Int J Mol Sci. 2023 Jun 24;24(13):10580. doi: 10.3390/ijms241310580 (PMC10341584; doi:10.3390/ijms241310580)
Supplement: Supplementary file 1 [file ijms-24-10580-s001.zip › SupplementaryFile.pdf]

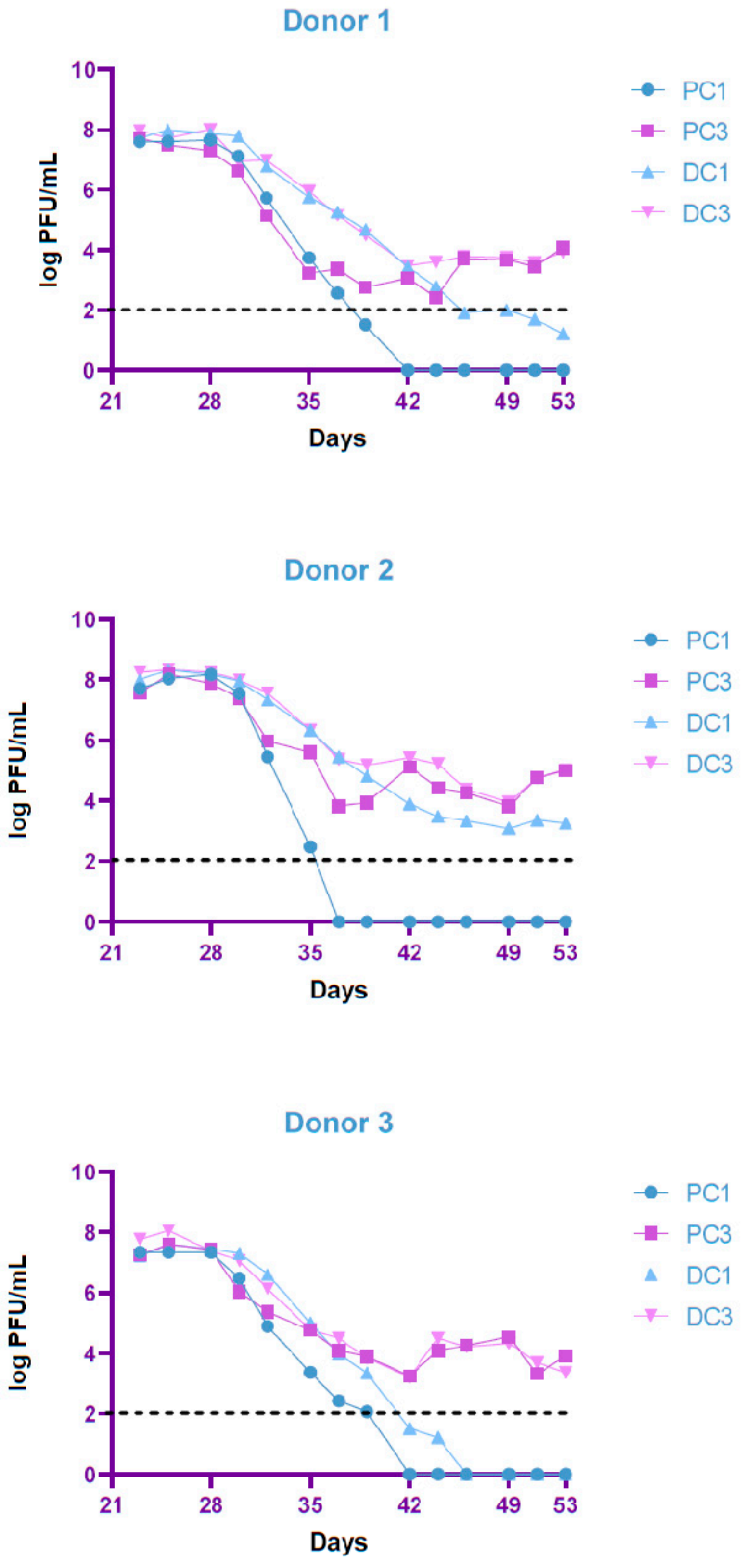

Figure S1: Individual values of phage K1_ULINTec4 concentrations measured. PC: proximal colon, DC: distal colon, 1: system 1 (phage alone), 3: system 3 (bacteria+phage). The dotted line represents the limit of quantification.

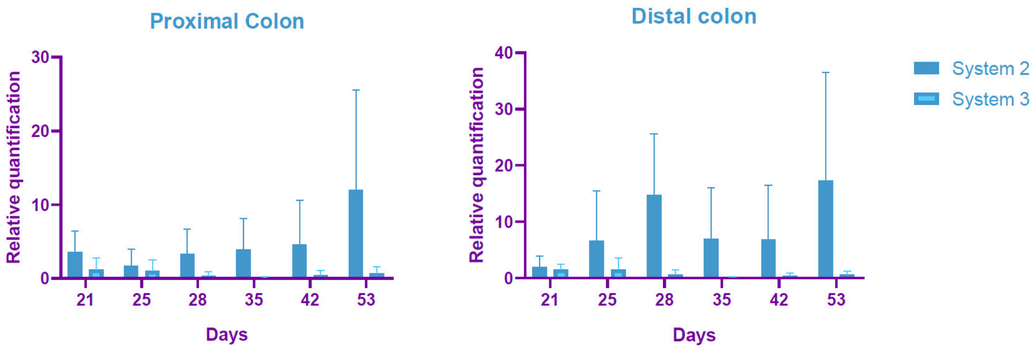

Figure S2: Comparisons of relative quantifications of *E. coli* K1 between systems 2 and 3. Data presented were calculated using $2^{-\Delta\Delta Cq}$ method. PC: proximal colon, DC: distal colon. The replicates represent the mean results of the three separate donors with SD. No statistically significant difference was highlighted.

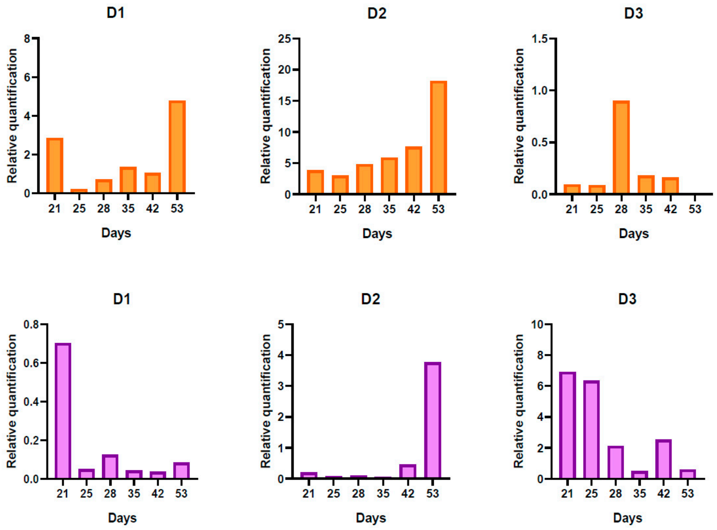

Figure S3: Individual values of relative quantifications of *E. coli* K1 in proximal colons. Orange: system 2, Purple: system 3. Data presented were calculated using $2^{-\Delta\Delta Cq}$ method. D1: donor 1, D2: donor 2, D3: donor 3

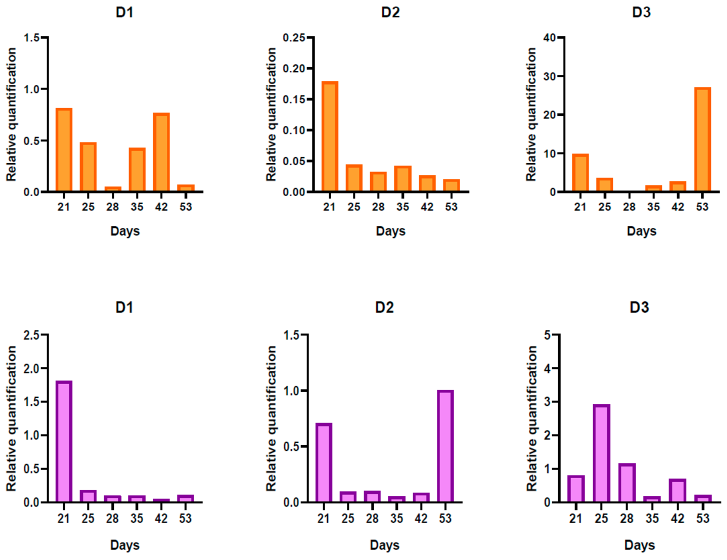

Figure S4: Individual values of relative quantifications of *E. coli* K1 in distal colons. Orange: system 2, Purple: system 3 Data presented were calculated using $2^{-\Delta\Delta Cq}$ method. D1: donor 1, D2: donor 2, D3: donor 3

Table S1. Evolution of Shannon, Simpson and Piélou alpha diversity indexes in proximal and distal colons of the three systems. Results of the three donors are presented as mean ± SD.

		Days							
		14	21	25	28	35	42	49	53
PC1	Shannon	1.26±0.08	1.39±0.14	1.21±0.26	1.18±0.31	1.41±0.02	1.36±0.04	1.41±0.17	1.54±0.2
	Simpson	0.55±0.08	0.62±0.14	0.54±0.18	0.55±0.22	0.62±0.07	0.58±0.06	0.6±0.12	0.63±0.03
	Piélou	0.44±0.08	0.5±0.13	0.43±0.14	0.42±0.15	0.48±0.09	0.49±0.05	0.48±0.1	0.52±0.02
PC2	Shannon	1.37±0.38	1.39±0.02	1.49±0.27	1.47±0.26	1.6±0.12	1.67±0.23	1.85±0.2	1.84±0.21
	Simpson	0.61±0.11	0.63±0.07	0.66±0.11	0.65±0.12	0.72±0.04	0.73±0.06	0.78±0.02	0.79±0.02
	Piélou	0.47±0.1	0.5±0.1	0.53±0.11	0.5±0.09	0.57±0.05	0.55±0.05	0.62±0.02	0.61±0.02
PC3	Shannon	1.21±0.19	1.61±0.22	1.55±0.06	1.43±0.16	1.44±0.32	1.34±0.42	1.63±0.41	1.64±0.32
	Simpson	0.53±0.14	0.7±0.03	0.71±0.04	0.66±0.1	0.66±0.13	0.6±0.21	0.72±0.12	0.72±0.12
	Piélou	0.45±0.15	0.54±0.05	0.54±0.08	0.5±0.15	0.51±0.14	0.46±0.17	0.55±0.13	0.55±0.13
DC1	Shannon	2.29±0.77	2.37±0.55	2.32±0.7	2.44±0.64	2.46±0.41	2.43±0.68	2.18±0.82	2.09±1.03
	Simpson	0.76±0.17	0.79±0.12	0.78±0.18	0.84±0.09	0.84±0.04	0.82±0.11	0.74±0.19	0.7±0.28
	Piélou	0.61±0.11	0.63±0.1	0.61±0.14	0.66±0.09	0.66±0.04	0.64±0.08	0.57±0.13	0.54±0.2
DC2	Shannon	2.29±0.44	2.26±0.35	2.32±0.22	2.4±0.22	2.53±0.39	2.52±0.47	2.11±0.57	1.92±0.55
	Simpson	0.81±0.06	0.8±0.1	0.82±0.06	0.85±0.03	0.87±0.03	0.85±0.06	0.76±0.14	0.68±0.17
	Piélou	0.62±0.08	0.62±0.13	0.62±0.07	0.64±0.07	0.67±0.03	0.66±0.04	0.6±0.1	0.52±0.14
DC3	Shannon	2.47±0.59	2.42±0.28	2.43±0.16	2.46±0.29	2.48±0.28	2.43±0.6	2.37±0.56	2.19±0.47
	Simpson	0.82±0.1	0.83±0.04	0.84±0.01	0.84±0.03	0.85±0.03	0.83±0.11	0.81±0.08	0.77±0.09
	Piélou	0.66±0.07	0.65±0.02	0.66±0.05	0.66±0.06	0.67±0.05	0.65±0.11	0.63±0.08	0.59±0.06

PC: proximal colon, DC: distal colon, 1: system 1, 2: system 2, 3: system 3

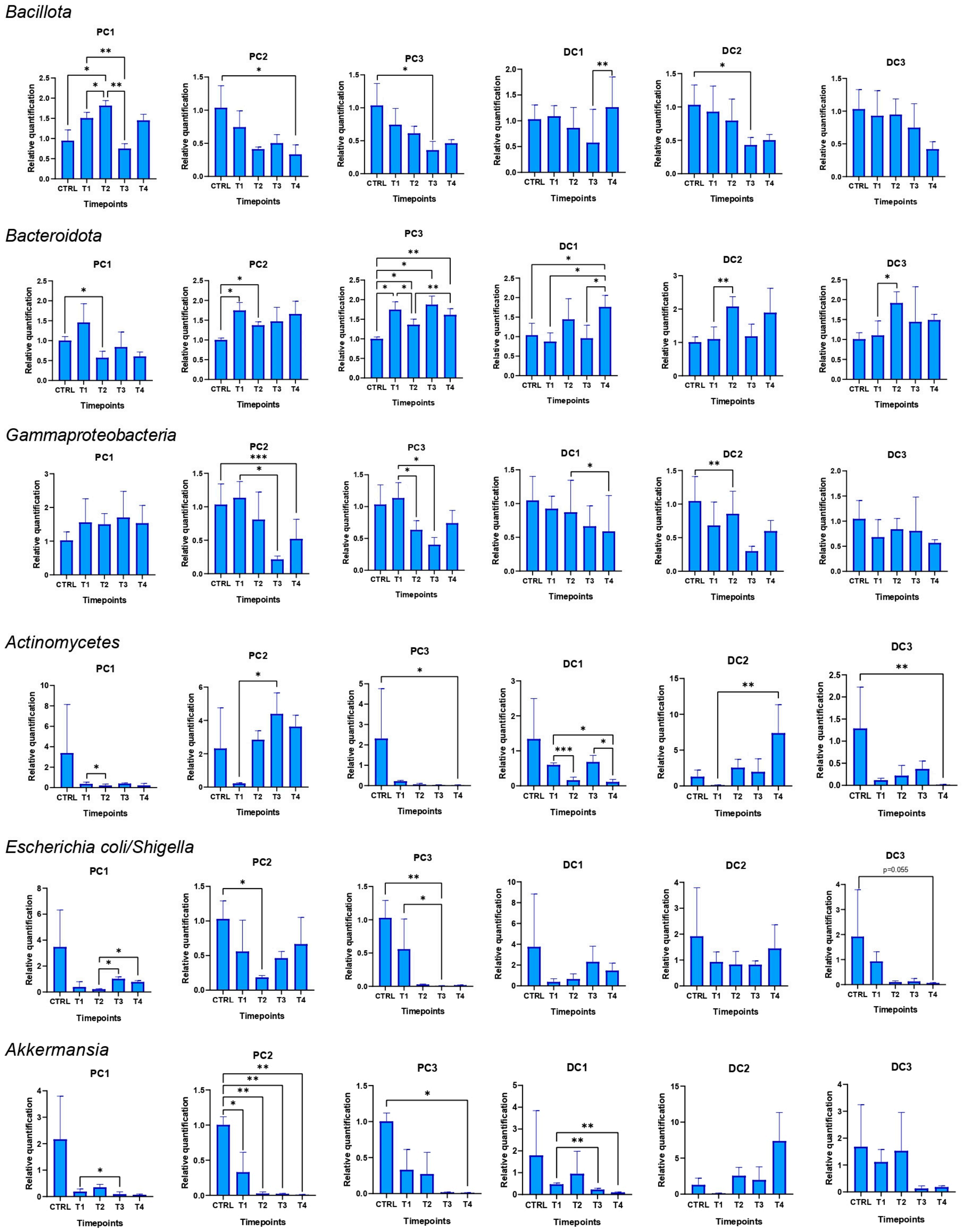

Figure S5 : Relative quantification of taxa assessed by qPCR from donor 1. Data presented were calculated using $2^{-\Delta\Delta Cq}$ method. PC: proximal colon, DC: distal colon, 1: system 1, 2: system 2, 3: system 3. Statistical significance is indicated as $p < 0.05$ (*), $p < 0.01$ (**).

Table S2. Summary of qPCR p-values from donor 1

Target	Bioreactor	Timepoints	p-value
Bacillota	PC1	CTRL-T2	0.0455
Bacillota	PC1	T1-T2	0.0359
Bacillota	PC1	T2-T3	0.0021
Bacillota	PC1	T1-T3	0.0022
Bacillota	DC1	T3-T4	0.0092
Bacillota	PC2	CTRL-T4	0.0365
Bacillota	PC3	CTRL-T3	0.0175
Bacillota	DC2	CTRL-T3	0.0175
Bacteroidota	PC1	CTRL-T2	0.0373
Bacteroidota	PC2	CTRL-T1	0.0140
Bacteroidota	PC2	CTRL-T2	0.0145
Bacteroidota	PC3	CTRL-T1	0.014
Bacteroidota	PC3	CTRL-T2	0.0248
Bacteroidota	PC3	CTRL-T3	0.0154
Bacteroidota	PC3	CTRL-T4	0.0096
Bacteroidota	PC3	T1-T2	0.0457
Bacteroidota	PC3	T2-T4	0.0088
Bacteroidota	DC1	CTRL-T4	0.0137
Bacteroidota	DC1	T1-T4	0.0107
Bacteroidota	DC1	T3-T4	0.0143
Bacteroidota	DC2	T1-T2	0.0014
Bacteroidota	DC3	T1-T2	0.0450
Gammaproteobacteria	PC2	CTRL-T4	0.0003
Gammaproteobacteria	PC2	T1-T3	0.0306
Gammaproteobacteria	PC3	T1-T2	0.0325
Gammaproteobacteria	PC3	T1-T3	0.0123
Gammaproteobacteria	DC1	T2-T4	0.0284
Gammaproteobacteria	DC2	CTRL-T2	0.0073
Actinomycetes	PC1	T1-T2	0.0148
Actinomycetes	PC2	T1-T3	0.0175
Actinomycetes	PC3	CTRL-T4	0.0365
Actinomycetes	DC1	T1-T2	0.0005
Actinomycetes	DC1	T1-T4	0.0197
Actinomycetes	DC1	T3-T4	0.0136
Actinomycetes	DC2	T1-T4	0.0035
Actinomycetes	DC3	CTRL-T4	0.0175
Escherichia coli/Shigella	PC1	T2-T3	0.0115
Escherichia coli/Shigella	PC1	T2-T4	0.0134
Escherichia coli/Shigella	PC2	CTRL-T2	0.0175
Escherichia coli/Shigella	PC3	CTRL-T3	0.008
Escherichia coli/Shigella	PC3	T1-T3	0.0365
Escherichia coli/Shigella	DC3	CTRL-T4	0.055
Akkermansia	PC1	T1-T3	0.0223
Akkermansia	PC2	CTRL-T1	0.0322
Akkermansia	PC2	CTRL-T2	0.0014
Akkermansia	PC2	CTRL-T3	0.0016
Akkermansia	PC2	CTRL-T4	0.0017
Akkermansia	PC3	CTRL-T4	0.0175
Akkermansia	DC1	T1-T3	0.0063
Akkermansia	DC1	T1-T4	0.0035

PC: proximal colon, DC: distal colon, 1: system 1, 2: system 2, 3: system 3.

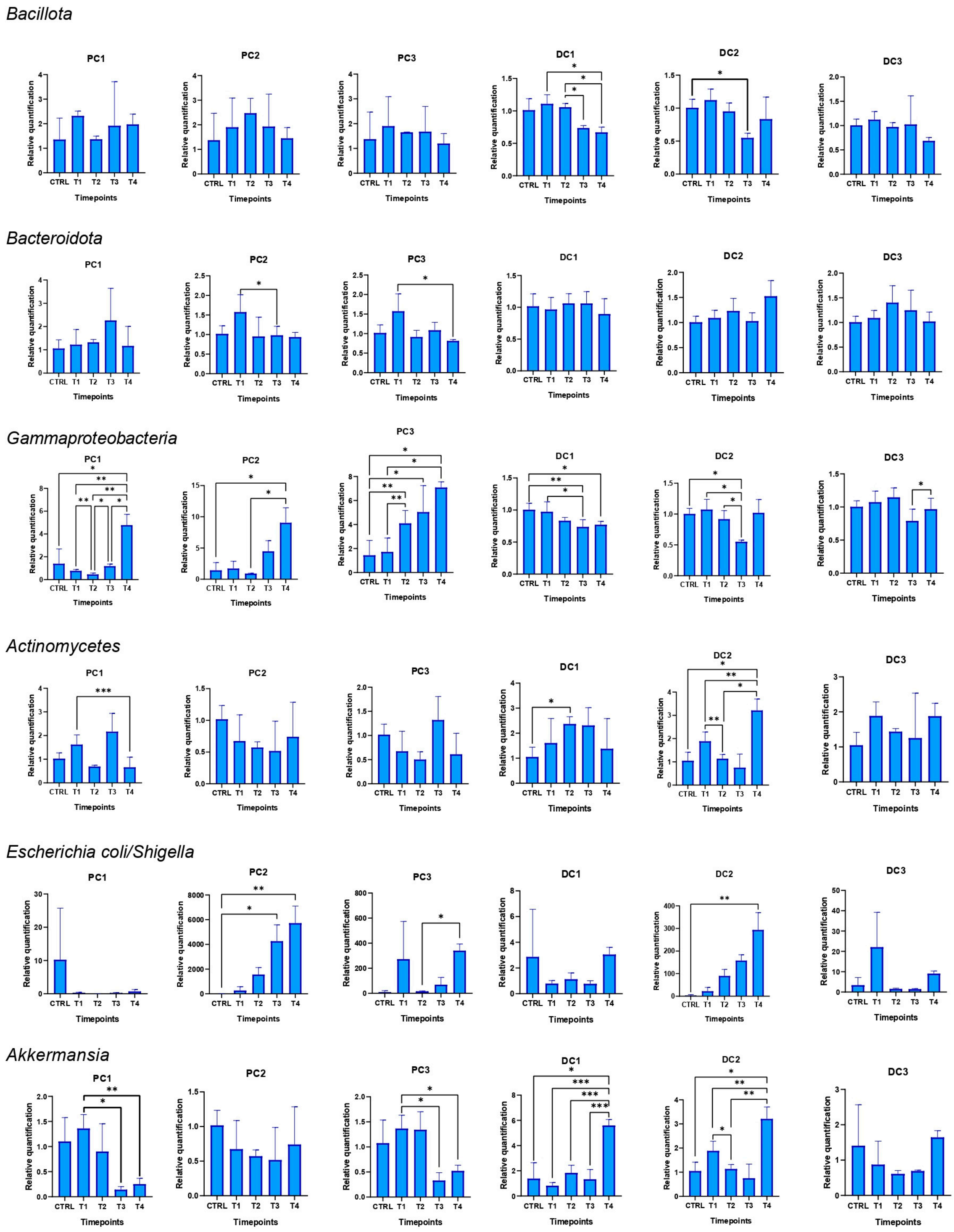

Figure S6 : Relative quantification of taxa assessed by qPCR from donor 2. Data presented were calculated using $2^{-\Delta\Delta Cq}$ method. PC: proximal colon, DC: distal colon, 1: system 1, 2: system 2, 3: system 3. Statistical significance is indicated as $p < 0.05$ (*), $p < 0.01$ (**), $p < 0.001$ (***).

Table S3. Summary of qPCR p-values from donor 2

Target	Bioreactor	Timepoints	p-value
Bacillota	PC1	T1-T2	0.0232
Bacillota	DC1	T1-T4	0.0421
Bacillota	DC1	T2-T3	0.016
Bacillota	DC1	T2-T4	0.0289
Bacillota	DC2	CTRL-T3	0.0413
Bacillota	DC3	CTRL-T4	0.0232
Bacillota	DC3	T1-T4	0.0320
Bacteroidota	PC2	T1-T3	0.0492
Bacteroidota	PC3	T1-T4	0.0365
Gammaproteobacteria	PC1	CTRL-T4	0.0489
Gammaproteobacteria	PC1	T1-T2	0.0087
Gammaproteobacteria	PC1	T1-T4	0.0094
Gammaproteobacteria	PC1	T2-T3	0.0409
Gammaproteobacteria	PC1	T2-T4	0.0086
Gammaproteobacteria	PC1	T3-T4	0.0203
Gammaproteobacteria	PC2	CTRL-T4	0.0175
Gammaproteobacteria	PC2	T2-T4	0.0365
Gammaproteobacteria	PC3	CTRL-T2	0.0044
Gammaproteobacteria	PC3	CTRL-T3	0.0395
Gammaproteobacteria	PC3	CTRL-T4	0.0256
Gammaproteobacteria	PC3	T1-T2	0.0017
Gammaproteobacteria	PC3	T1-T4	0.0268
Gammaproteobacteria	DC1	CTRL-T3	0.0033
Gammaproteobacteria	DC1	CTRL-T4	0.0315
Gammaproteobacteria	DC1	T1-T3	0.0283
Gammaproteobacteria	DC2	CTRL-T3	0.0135
Gammaproteobacteria	DC2	T1-T3	0.0359
Gammaproteobacteria	DC2	T2-T3	0.0322
Gammaproteobacteria	DC3	T3-T4	0.0110
Actinomycetes	PC1	T1-T4	<0.0001
Actinomycetes	DC1	CTRL-T2	0.0260
Actinomycetes	DC2	CTRL-T4	0.0465
Actinomycetes	DC2	T1-T2	0.0337
Actinomycetes	DC2	T1-T4	0.0035
Actinomycetes	DC2	T2-T4	0.0044
Escherichia coli/Shigella	PC2	CTRL-T3	0.0365
Escherichia coli/Shigella	PC2	CTRL-T4	0.008
Escherichia coli/Shigella	PC3	T2-T4	0.0365
Escherichia coli/Shigella	DC1	T1-T4	0.0070
Escherichia coli/Shigella	DC1	T3-T4	0.0250
Escherichia coli/Shigella	DC2	CTRL-T4	0.0035
Akkermansia	PC1	T1-T3	0.0214
Akkermansia	PC1	T1-T4	0.0041
Akkermansia	PC3	T1-T3	0.0294
Akkermansia	PC3	T1-T4	0.0094
Akkermansia	DC1	CTRL-T4	0.0123
Akkermansia	DC1	T1-T4	0.0003
Akkermansia	DC1	T2-T4	0.0003
Akkermansia	DC1	T3-T4	0.0005
Akkermansia	DC2	CTRL-T4	0.0465
Akkermansia	DC2	T1-T2	0.0337
Akkermansia	DC2	T1-T4	0.0035
Akkermansia	DC2	T2-T4	0.0044

PC: proximal colon, DC: distal colon, 1: system 1, 2: system 2, 3: system 3.

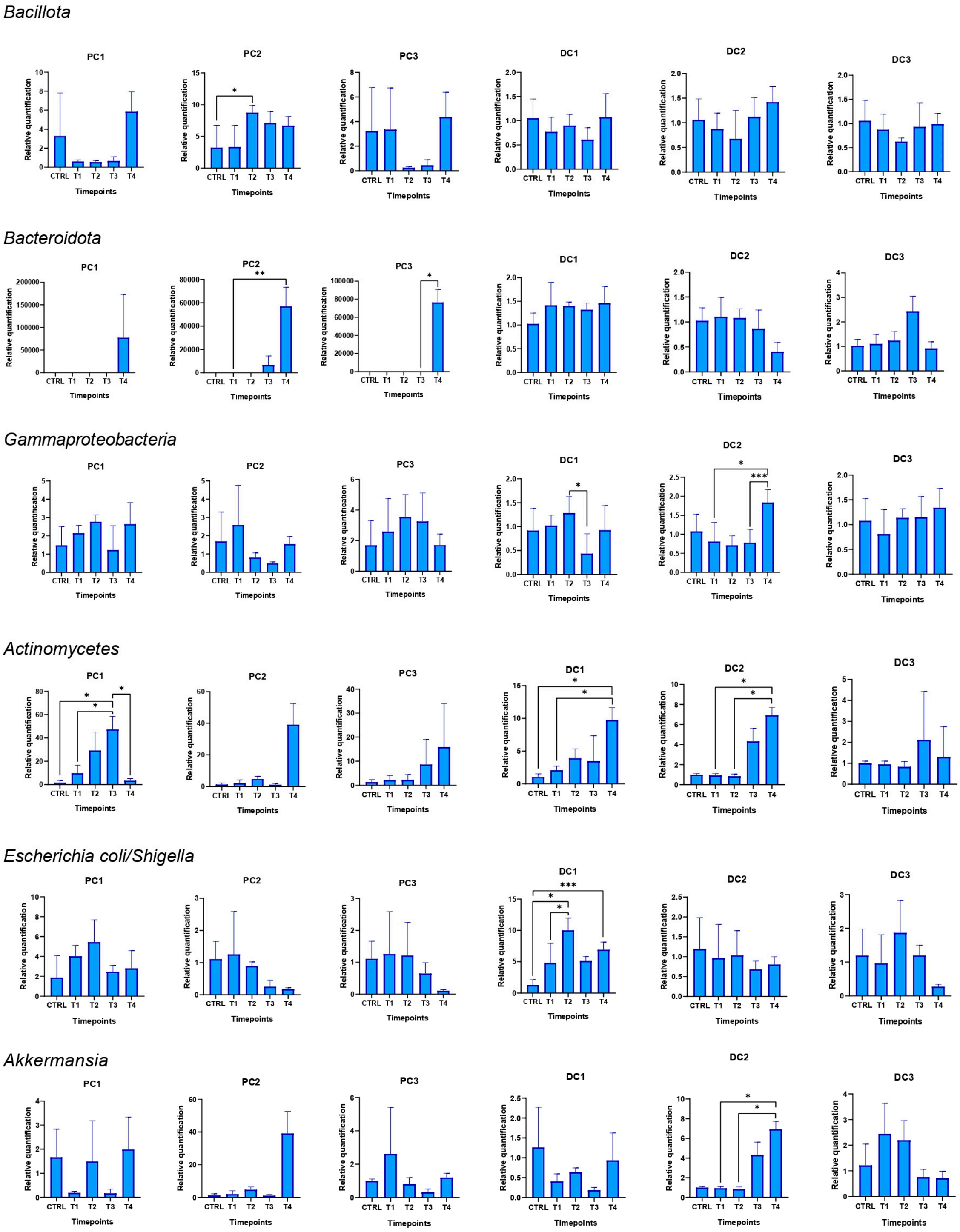

Figure S7 : Relative quantification of taxa assessed by qPCR from donor 3. Data presented were calculated using $2^{-\Delta\Delta Cq}$ method. PC: proximal colon, DC: distal colon, 1: system 1, 2: system 2, 3: system 3. Statistical significance is indicated as $p < 0.05$ (*), $p < 0.01$ (**), $p < 0.001$ (***).

Table S4: Summary of qPCR p-values from donor 3

Target	Bioreactor	Timepoints	p-value
Bacillota	PC1	T1-T4	0.0499
Bacillota	PC2	CTRL-T2	0.017
Bacteroidota	PC2	T1-T4	0.008
Bacteroidota	PC3	T3-T4	0.0175
Gammaproteobacteria	PC1	CTRL-T4	0.0365
Gammaproteobacteria	DC1	T2-T3	0.0187
Gammaproteobacteria	DC2	T1-T4	0.0137
Gammaproteobacteria	DC2	T3-T4	0.001
Actinomycetes	PC1	CTRL-T3	0.0145
Actinomycetes	PC1	T1-T3	0.0264
Actinomycetes	PC1	T3-T4	0.0207
Actinomycetes	DC1	CTRL-T4	0.0107
Actinomycetes	DC1	T1-T4	0.018
Actinomycetes	DC2	T1-T4	0.0365
Actinomycetes	DC2	T2-T4	0.0365
Escherichia coli/Shigella	DC1	CTRL-T2	0.0224
Escherichia coli/Shigella	DC1	CTRL-T4	0.0004
Escherichia coli/Shigella	DC1	T1-T2	0.0466
Akkermansia	DC1	T2-T3	0.0059
Akkermansia	DC2	T1-T4	0.0365
Akkermansia	DC2	T2-T4	0.0365

PC: proximal colon, DC: distal colon, 1: system 1, 2: system 2, 3: system 3.
